# SNPase-ARMS qPCR: Ultrasensitive Mutation-Based Detection of Cell-Free Tumor DNA in Melanoma Patients

**DOI:** 10.1371/journal.pone.0142273

**Published:** 2015-11-12

**Authors:** Julia Stadler, Johanna Eder, Barbara Pratscher, Sabine Brandt, Doris Schneller, Robert Müllegger, Claus Vogl, Franz Trautinger, Gottfried Brem, Joerg P. Burgstaller

**Affiliations:** 1 Biotechnology in Animal Production, Department for Agrobiotechnology, IFA Tulln, Tulln, Lower Austria, Austria; 2 Institute of Animal Breeding and Genetics, University of Veterinary Medicine Vienna, Vienna, Austria; 3 Department of Dermatology and Venereology, Karl Landsteiner University of Health Sciences, St. Poelten, Lower Austria, Austria; 4 Karl Landsteiner Institute of Dermatological Research, St. Poelten, Lower Austria, Austria; 5 Research Group Oncology of the Equine Clinic, Department for Companion Animal and Horses, University of Veterinary Medicine Vienna, Vienna, Austria; 6 Department of Dermatology and Venereology, Landesklinikum Wiener Neustadt, Wiener Neustadt, Lower Austria, Austria; Universidade de São Paulo, BRAZIL

## Abstract

Cell-free circulating tumor DNA in the plasma of cancer patients has become a common point of interest as indicator of therapy options and treatment response in clinical cancer research. Especially patient- and tumor-specific single nucleotide variants that accurately distinguish tumor DNA from wild type DNA are promising targets. The reliable detection and quantification of these single-base DNA variants is technically challenging. Currently, a variety of techniques is applied, with no apparent “gold standard”. Here we present a novel qPCR protocol that meets the conditions of extreme sensitivity and specificity that are required for detection and quantification of tumor DNA. By consecutive application of two polymerases, one of them designed for extreme base-specificity, the method reaches unprecedented sensitivity and specificity. Three qPCR assays were tested with spike-in experiments, specific for point mutations *BRAF* V600E, *PTEN* T167A and *NRAS* Q61L of melanoma cell lines. It was possible to detect down to one copy of tumor DNA per reaction (Poisson distribution), at a background of up to 200 000 wild type DNAs. To prove its clinical applicability, the method was successfully tested on a small cohort of *BRAF* V600E positive melanoma patients.

## Introduction

Patient-specific biomarkers that serve as indicators of therapy options and treatment response are rapidly gaining importance in clinical cancer treatment. Especially cell-free circulating DNA (cfDNA) has become a common point of interest. cfDNA are small fragments of nucleic acids in the peripheral blood circulation, supposed to be actively released by living cells [1], but also deriving from apoptotic and necrotic processes. Consequently, in pathological conditions like cancer (but also trauma and inflammation) cfDNA levels can significantly increase. The processes that lead to higher levels of cfDNA during cancer development and dissemination are still poorly understood. Plasma concentrations of cfDNA can vary widely between 0 and 100 ng per milliliter in healthy individuals [2] and up to 1000 ng per milliliter in cancer patients [3].

In patients with (metastatic) cancer, the portion of cfDNA that is tumor-derived is referred to as circulating tumor DNA (ctDNA) [4]. Plasma can serve as “liquid biopsy” to monitor the changes of ctDNA yields during the course of the disease and the efficiency of anticancer therapies [5]. Several studies have shown that ctDNA in the peripheral blood of patients in an advanced stage of disease holds great potential as prognostic and predictive biomarker [1, 2, 6].

To utilize ctDNA as a biomarker, it is necessary to distinguish tumor DNA from non-mutated wild type DNA. This is achieved by detection of genetic aberrations, e.g., rearrangements, chromosomal copy number changes, and point mutations (SNV, single nucleotide variant), with SNVs being the most abundant [4]. Tumor cells accumulate numerous mutations over time, approximately at the same rate as normal cells [7]. Tumor progenitor cells divide rapidly [8] and acquire SNVs in many genes, only some of these inducing tumors [9, 10]. Due to the clonal expansion of the tumor, these SNVs are present in virtually every tumor cell [11, 12]. Consequently, SNVs can be elucidated by sequencing both, healthy and tumor tissue of the patient [11]. Therefore the detection and quantification of tumor specific SNVs in the plasma of a patient is equivalent to the detection of ctDNA. The major technical challenge to using SNVs as biomarkers is reliable detection. When present, ctDNA is expected to represent only a minor fraction of less than 0.01% of the total circulating DNA [11]. Thus detecting SNVs can be compared to “finding the needle in a haystack” [13].

Currently, many techniques for detecting SNVs are being used, all showing several advantages and disadvantages; but there exists no apparent “gold standard”. These approaches include next generation sequencing (NGS), digital PCR (dPCR), BEAMing and (allele specific) qPCR. An overview of the most prominent methods is given in Table 1. The lack of a standard compromises comparisons between different methods as regards the critical condition of low tumor copy number against a background of large numbers of wild type DNA. Generally, traditional methods show sensitivity thresholds of only approximately 1% [14], whereas more recent, technically demanding methods reach sensitivities of 1 SNV in 20 000 wild type DNAs [4, 11], with a maximum of 1 in 100 000 [15].

**Table 1:** Overview of methods for cfDNA detection in plasma of cancer patients.

Type of tumor	Technique	Sensitivity^1^	Gene	References
**Melanoma**	dPCR	0.005%	BRAF	[16]
	dPCR	0.001%	BRAF	[17]
	ARMS qPCR	0.1%	BRAF	[18]
	ARMS qPCR	0.1%	BRAF	[19]
	AS qPCR	0.3%	BRAF	[20]
	COLD PCR	3.1%	BRAF	[21]
	LNA qPCR	0.3%	BRAF	[22]
	PNA LNA qPCR	0.001%	BRAF	[23]
**CRC** ^2^	BEAMing	≥ 0.01%	APC	[11, 24]
	Intplex (AS qPCR)	0.004–0.014%	KRAS, BRAF	[25]
	Intplex (AS qPCR)	≥ 0.005%	KRAS, BRAF	[26]
	LNA qPCR	0.01%	BRAF	[27]
**NSCLC** ^3^	CAPP-Seq	0.025%	ALK, ROS1, RET	[28]
	dPCR	0.05–0.5%	EGFR, KRAS, BRAF	[29]
	BEAMing	0.01%	EGFR	[30]
	DISSECT (PNA LNA qPCR)	0.01%	EGFR	[31]
**Breast cancer**	dPCR / targeted deep sequencing	0.1% / 0.14%	PIK3CA, TP53	[32]
	dPCR	0.01–2.99%	PIK3CA	[33]
	BEAMing	≥ 0.01%	PIK3CA	[34]
	AS qPCR	0.1–1%	PIK3CA	[35, 36]

^1^mutant to wild type ratio

^2^CRC: colorectal carcinoma

^3^NSCLC: non-small cell lung cancer

What are the requirements for a successful approach to detect SNVs? (i) The method needs high sensitivity and specificity, i.e. has to be able to detect mutated DNA within a vast number of non-mutated DNA. (ii) Due to the varying copy numbers of ctDNA in the plasma of cancer patients, detection needs to be possible over a wide range, from thousands of copies down to as little as one copy per sample. (iii) The method has to be routinely applicable, i.e. time, costs, technical equipment and the training requirements of the personnel have to be considered. Currently, no method fulfils all these requirements.

**Fig 1:**
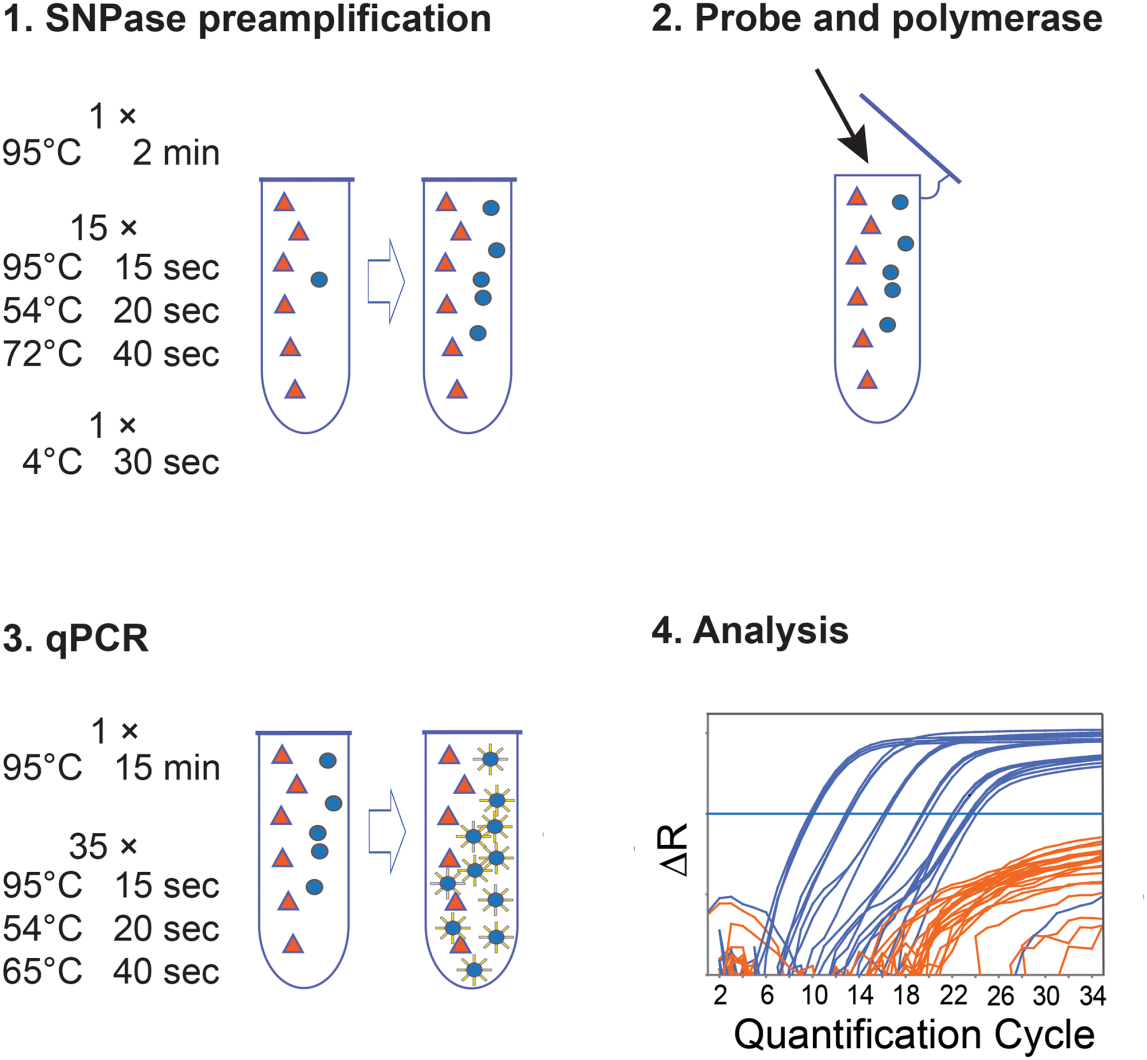
Workflow of SNPase-ARMS qPCR. Fig 1 shows the workflow of SNPase-ARMS qPCR. 1. SNPase preamplification: with the SNPase polymerase, allele-specific primers amplify the target DNA based on the respective single nucleotide variant (SNV) with extreme sensitivity. In 15 PCR cycles the ratio between target (blue circles) and non-target (orange triangles) DNA is changed towards the target DNA. An exemplary temperature protocol for the *BRAF* V600E assay is shown. The last PCR cycle ends in a 4°C step to inhibit unspecific elongation. The PCR plate is put on ice immediately afterwards, and kept on ice during the next step. 2. Probe and Polymerase: the reaction tube (PCR plate) is opened (preferentially in a separate room to avoid contamination), and 5′ to 3′ exonuclease active polymerase and hydrolysis probe are added. 3. qPCR: in this step, the already preamplified target gene is amplified by the 5′ to 3′ exonuclease active polymerase. The initial step, 95°C for 15 minutes, inhibits the residual SNPase polymerase, and activates the newly added hot-start polymerase. During the following standard qPCR, the sequence-specific hydrolysis probe is cleaved and a fluorescence signal corresponding to the number of cleaved probes is created (symbolized by blue circles with a yellow corona). An exemplary temperature protocol for the *BRAF* V600E assay is shown. 4. Analysis: the qPCR is evaluated via the amplification plot. Quantification of positive samples is performed with the standard curve method [37] using the ViiA^™^ Software, v1.2.4.

Here we present a novel, highly sensitive allele-specific qPCR protocol, for which we propose the term SNPase-ARMS qPCR. It is based on the consecutive application of two different polymerases. The first polymerase is optimized for point mutations detection, albeit without 5′ to 3′ exonuclease activity. In a pre-amplification step, it provides specificity by selectively amplifying target-DNA and preventing amplification of non-target DNA. The second polymerase amplifies the (pre-amplified) DNA, adding with its 5′ to 3′ exonuclease activity the sensitivity and specificity of dual-labelled hydrolysis probes (Fig 1). By using this protocol, a sensitivity of 1 mutated DNA in 200 000 wild type DNAs can be achieved. The approach can generally be adapted to all SNVs. We have successfully tested qPCR assays in spiking experiments for *BRAF* V600E, *PTEN* T167A and *NRAS* Q61L. To prove the clinical applicability of our method, we tested the plasma of a small cohort of *BRAF* V600E positive melanoma patients and found the respective mutation in all patients with metastatic cancer.

## Methods

### Ethics Statement

The survey was approved by the regional ethics committee of Lower Austria (GS4-EK-4/213-2013). Written informed consent was obtained from all participants.

### Cell culture

Melanoma cell lines A375, SKMel28 and MelJuso (first description [38–41]) carrying homozygous *BRAF* V600E, *PTEN* T167A and *NRAS* Q61L mutation were used as source for tumor DNA for tests of the assays. The respective correspondence between cell lines and mutations is as follows: A375 and SKMel28 for *BRAF* V600E, SKMel28 for *PTEN* T167A and MelJuso for *NRAS* Q61L [42]. All cell lines utilized in this study were kindly provided by Prof. Dr. Hubert Pehamberger (Department of Dermatology, Medical University of Vienna, Austria). A375 and SKMel28 cells were originally purchased from the American Type Culture Collection (ATCC, LGC Promochem GmbH, Wesel, Germany) [43, 44], whereas MelJuso cells were originally acquired from the Leibniz Institute DSMZ, German Collection of Microorganisms and Cell Cultures (Leibniz-Institut DSMZ, Braunschweig, Germany) [43]. The melanoma cell lines were cultivated in Dulbecco’s Modified Eagle’s Medium (GE Healthcare, Austria) containing 10% fetal bovine serum (Gibco^®^, Life Technologies) and 1% penicillin/ streptomycin (Gibco^®^, Life Technologies). Melanoma cells were cultured at 37°C in a 5% CO_2_ humidified atmosphere. Cells were grown until at least 70% confluence in T 75 cm^2^ flasks (Greiner Bio-One, Austria) and passaged twice a week according to standard procedures.

### PBMC isolation

30 ml whole blood from healthy donors was layered over 15 ml density gradient medium (Ficoll-Paque, GE Healthcare, Austria) in 50 ml tubes (SepMate, Stemcell Technologies, France). After a centrifugation step at 1200 g for 20 min, the interphase was transferred to new tubes and diluted to 50 ml with 1 × PBS (GE Healthcare, Austria). Subsequently, centrifugation at 1200 g for 10 min was carried out, the supernatant being discarded. Following a final washing step, PBMC pellets were resuspended in 200 μl 1 × PBS (GE Healthcare, Austria) and stored at -20°C until further processing.

### DNA extraction

Genomic DNA (gDNA) from melanoma cell lines and PBMC pellets were extracted using Qiagen DNeasy Blood & Tissue Kit (Qiagen, Germany) according to the manufacturer's instructions. DNA from melanoma cell lines and PBMC pellets was eluted in 100 μl and 50 μl, respectively, of AE buffer. To obtain large volumes with high concentrations of PBMC gDNA, eluates were pooled. Quantity and quality of the extracted DNA were assessed using the NanoDrop Spectrometer 2000 (NanoDrop, Thermo Fisher Scientific).

### Clinical samples

From July 2013 to September 2014, nine *BRAF* V600E mutant melanoma patients were enrolled in this study. They were recruited at the Department of Dermatology, Karl Landsteiner University of Health Sciences, St. Poelten, Lower Austria. Blood samples were collected during routine care. *BRAF* V600E positive mutation status was evaluated using the *BRAF* V600E StripAssay (ViennaLab Diagnostics, Austria) via PCR amplification and was carried out in tissue (primary tumor or metastasis) in the context of standard management care of melanoma patients. Blood was also drawn from healthy control subjects in an Austrian blood donor center. Blood samples (30 ml each) were obtained by venipuncture using 6 ml sized K-EDTA blood collection tubes (Vacuette^®^ Blood Collection Tube), stored at room temperature, and processed within 2 hours of collection.

### Plasma isolation and cfDNA extraction

Plasma of patients and healthy donors was obtained after Ficoll-Paque (GE Healthcare, Austria) density gradient centrifugation of whole blood. The upper phase was transferred to 2 ml tubes and centrifuged at 16.000 g for 10 min at room temperature. Plasma samples were stored at -80°C until further analysis. cfDNA was isolated from 4 mL and 5 mL plasma of patients and healthy donors using the QIAamp circulating nucleic acid kit (Qiagen, Germany) according to the manufacturer instructions and eluted in a final volume of 30 μl Qiagen AVE buffer. Quantity and quality of the isolated DNA were assessed using the NanoDrop Spectrometer 2000 (NanoDrop, Thermo Fisher Scientific).

### SNV detection

SNV detection was based on a combination of preamplification with a polymerase specifically designed for detection of point mutations (SNPase, Bioron, Germany) and amplification refractory mutation system (ARMS) qPCR with a second polymerase with 5′ to 3′ exonuclease activity (Hot Firepol polymerase (Medibena Life Science, Austria)) and dual-labelled hydrolysis probes.

#### Oligonucleotide design

Three ARMS assays were designed for SNPase-ARMS qPCR using dual-labelled hydrolysis probes. ARMS primers were designed to detect the *BRAF* V600E, *PTEN* T167A and *NRAS* Q61L mutations. Further, for internal DNA amplification control in clinical samples, a MIA (melanoma-inhibiting activity) assay was designed. MIA is coded for by a single copy gene [45]. Primers and hydrolysis probes were designed with Primer Express Software v2.0 (Applied Biosystems, USA) and purchased from Sigma Aldrich (Germany) (for sequences see Table 2). ARMS-primers were designed with the SNV in question at the 3′-end. Moreover, to further increase specificity, an additional mismatch was included at the antepenultimate nucleotide [46]. All sequences were tested for intermolecular and self-molecular annealing (mfold, web server for nucleic acid folding [47]). Local alignment analyses were performed using the BLAST program to confirm the specificity of the designed primers [48].

**Table 2:** Primer and hydrolysis probe sequences.

Gene^1^	Forward primer^2^	Hydrolysis probe	Reverse primer^2^	Amplicon size (bp)	Primer / Probe Tm
**BRAF** [NC_000007.14]	GATTTTGGTCTAGCTACCG**A**	[6FAM]TCCCATCAGTTTGAACAGTTGTCTGG[BHQ1]	CTCAATTCTTACCATCCACAA	93	54 / 65
**PTEN** [NC_000010.11]	ACGACCCAGTTACCATAGCAAT	[6FAM]TGGCTTCTCTTTTTTTTCTGTCCACCAGGG[BHQ1]	GCCTCTGACTGGGAATCG**C**	113	61 / 72
**NRAS** [NC_000001.11]	ATACTGGATACAGCTGGCC**T**	[6FAM]CAGTGCCATGAGAGACCAATACATGAG[BHQ1]	GCAAATGACTTGCTATTATTGA	110	58 / 70
**MIA** [NC_000019.10]	GAGTGCAGCCGTAAGAAT	[6FAM]CATTCCCCTTCTATTCCTTCCCTAGACCC[BHQ1]	GCCACAGCCATGGAGATA	113	50 / 65

^1^ GenBank (NCBI) accession number in brackets.

^2^ 3′-prime allele-specific bases of ARMS primers are indicated by bold and underlined letters, intentional, additional mismatches to increase specificity are underlined.

Tm: melting temperature, °C; bp: base pair

#### SNPase-ARMS qPCR protocol

See Fig 1 for method workflow. 2 μl of DNA were amplified in a 25 μL-reaction volume containing 3.5 mM MgCl_2_ (Medibena Life Science, Austria), 200 nM deoxynucleotide triphosphates (Medibena Life Science, Austria), 300 nM of primer sets, 5 μl of 5 × SNPase buffer (Bioron, Germany), 5 units of SNPase Hotstart Polymerase (Bioron, Germany) and 9.95 μl MilliQ water. The first thermal protocol (preamplification) consisted of an initial denaturation step for 2 min at 95°C, followed by 15 cycles of 15 s at 95°C, 20 s at the respective assay specific annealing temperature (Table 2) and 40 s at 72°C. Preamplification was concluded by a single step of 4°C for 30 s. After that, the PCR plate was immediately stored on ice. For qPCR, 1 unit Hot Firepol polymerase (Medibena Life Science, Austria) and 100 nM hydrolysis probe were then added to each reaction with an electronic dispenser (Multipette^®^ stream, Eppendorf, Germany). Immediately afterwards, an initial activation of DNA polymerase (and inhibition of the SNPase polymerase) for 15 min at 95°C, followed by 35 cycles of 15 s at 95°C, primer annealing temperature for 20s and 40s at hydrolysis probe annealing temperature (Table 2), was carried out. To avoid contamination, this step was performed in a separate room. All steps were performed on a ViiA™ 7 qPCR System (Life Technologies, Germany). All reactions were run on 96 well plates (Life Technologies, Germany). Analysis was performed with the standard curve method [37] using the ViiA^™^ Software, v1.2.4.

#### Assay test experiments

**Fig 2:**
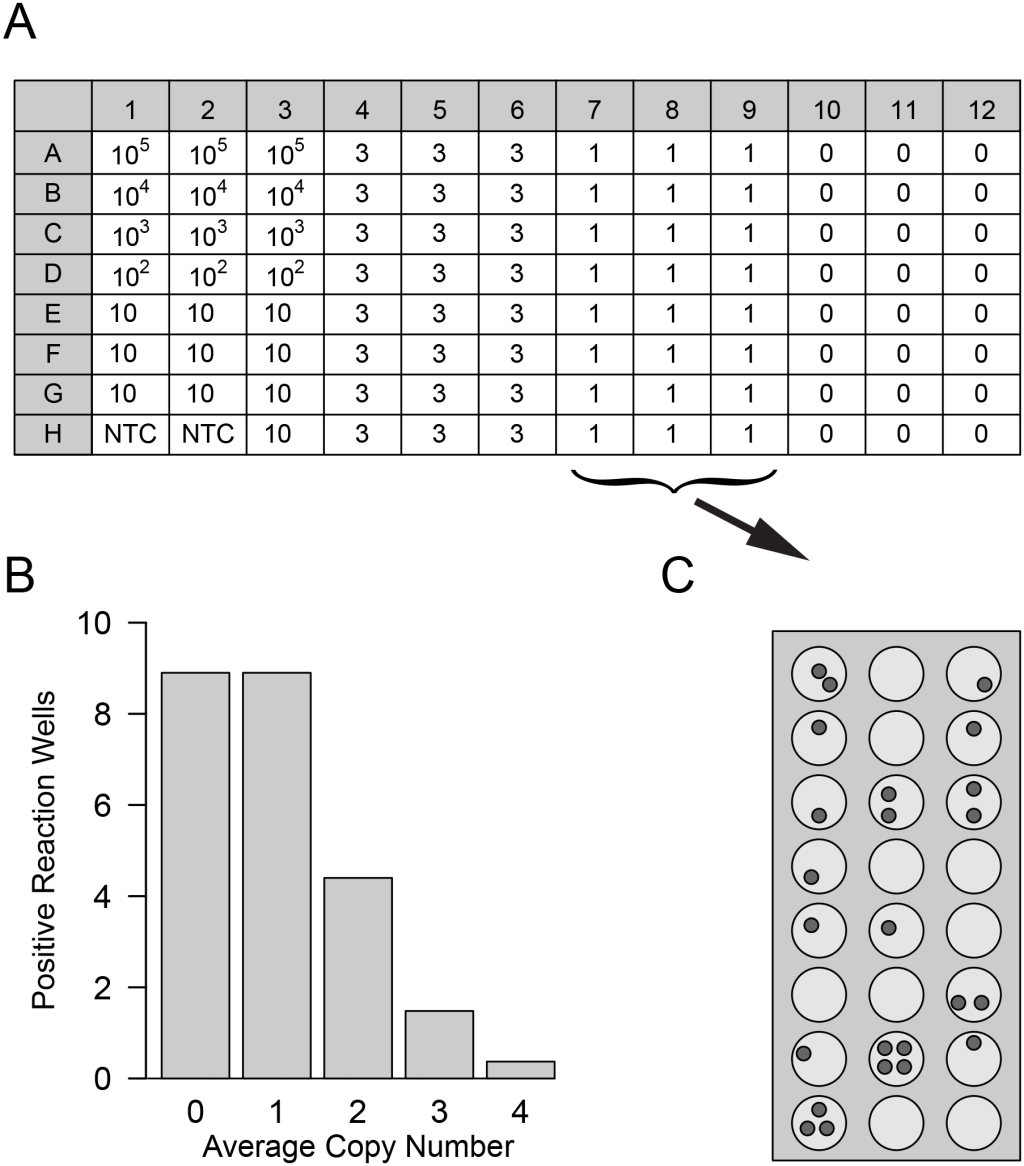
Target gene spike-in to test assay sensitivity. (A) Layout of 96-well microtiter plate to test for assay sensitivity, modified after Rossmanith and Wagner [49]. Spiked-in target gene copy numbers per reaction well are indicated at the respective locations. Wells containing 10^5^ to 100 copies were run in triplicate and used as standards in the analysis; samples of ten copies per well were analyzed in ten reactions; samples of three, one and 0 mutant copies in 24 reactions each. No-template controls (NTC) were included in duplicate. Each plate was repeated at least three times per assay. (B) At very low copy numbers, only part of the reaction wells can contain the target gene due to Poisson distribution. Therefore, even under ideal conditions less than 100% of the reactions can be positive. In this experiment 24 wells per plate were spiked to contain on average one target copy (plate columns 7–9). Due to Poisson distribution, the reaction wells are expected to contain from 0 to four target copies per well (as opposed to a single copy per well which represents the average), indicated on the x-axis of the bar chart. The y-axis indicates the predicted number out of 24 reaction wells that contain the respective copy number shown on the x-axis. This distribution is exemplarily depicted in (C), with small grey circles symbolizing target copies in the respective reaction wells.

Assay performance was tested with serial dilutions of tumor DNA derived from cell culture cells, against a defined background of wild type DNA from PBMCs. gDNA extracted from melanoma cell lines was diluted from 10^5^ copies per reaction to one copy as indicated in Fig 2. Calculation was based on the amount of 6 pg DNA per 2n nucleus, with cells being homozygous for the respective SNV. gDNA extracted from PBMCs at a concentration of 300 ng and 600 ng, equalling 10^5^ and 2 × 10^5^ wild type copies of the respective SNV, served as constant wild type DNA background.

Plate layout for all assay test experiments is shown in Fig 2. Standards were run in triplicate, spike-in samples of 10 copies per well were analyzed in 10 reactions, that of three and one copies as well as wild type control DNA were analyzed in 24 reaction wells. No template controls (NTC) were included in duplicate. Each assay was repeated three or five times.

### Analysis of clinical samples

cfDNA of plasma samples from nine *BRAF* V600E positive patients was analyzed with the respective assay. All plasma samples also underwent qPCR amplification of the MIA amplicon to confirm the presence of amplifiable DNA. One blood sample was obtained from each patient (except from patient number 6, who provided two blood samples) and analyzed in two independent qPCR runs. In each case, DNA from plasma samples from healthy donors was used as negative control. DNA from melanoma cell line A375 was used as a positive control. *BRAF* V600E quantification was calculated with an external standard curve [20, 50] derived from the test experiments.

### Statistical analysis

For statistical analysis of qPCR results, two parameter ranges need to be distinguished, a low range, where most of the variation comes from Poisson sampling and a high range, where the variance is supposed to rather scale with the square of the mean. In our case, interest focuses mostly on the low parameter range and, especially, on distinguishing between absences of tumor DNA from the presence of a low number of copies. With our data set, a square-root transformation of the quantification cycle (Cq) values removed most of the dependency of the variance on the mean.

#### Discrimination between positive and negative calls

**Fig 3:**
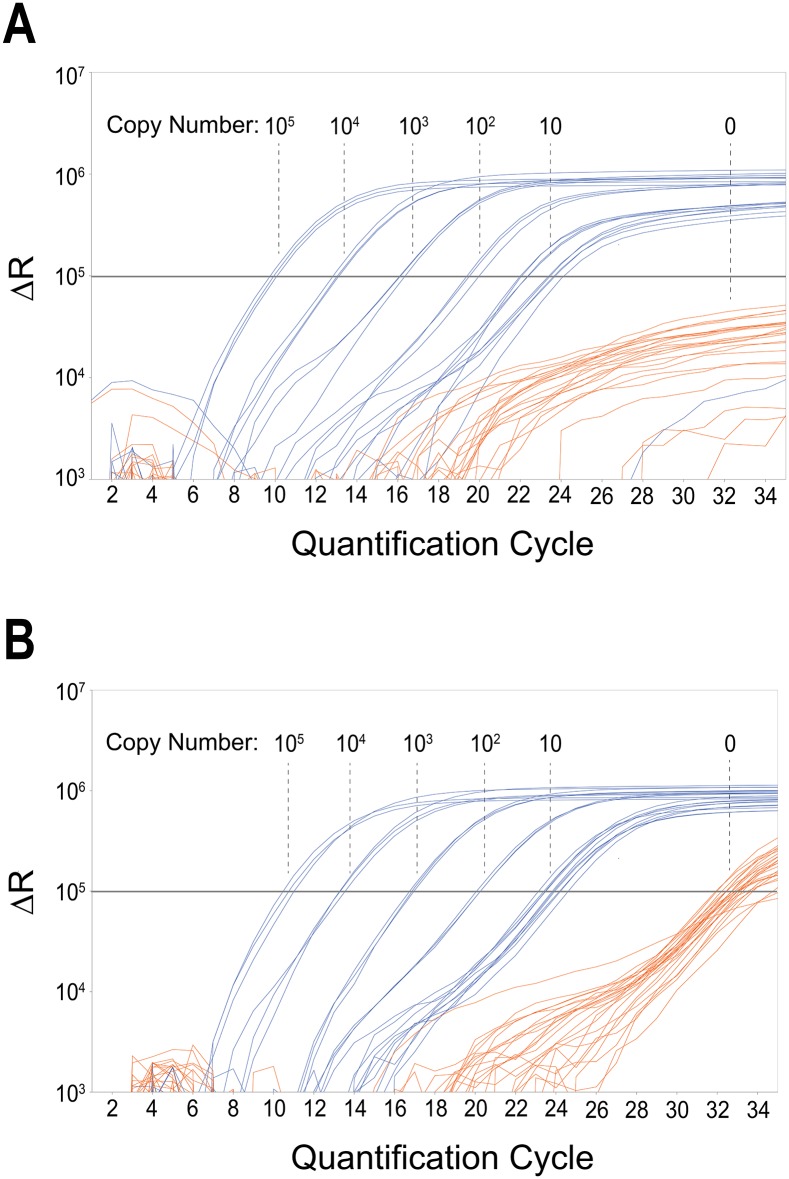
Dynamic range of *BRAF* V600E SNPase-ARMS qPCR. Exemplary qPCR amplification plots of a serial dilution of 10^5^ to ten *BRAF* V600E copies in a background of 2 × 10^5^ (A) and 10^5^ (B) wild type BRAF copies (following a 15 cycle SNPase preamplification step) are shown. The respective target-copy number is indicated in the plot. Delta R (y-axis) is plotted against quantification cycle (x-axis). qPCR threshold level is represented by the grey horizontal line. All reactions containing target DNA (blue) are positive and quantifiable with a single negative at ten copies in (A). Negative control samples (orange) show delayed amplification of approximately seven quantification cycles or do not amplify at all. No signal amplification was observed in the NTC sample wells. Results of wells containing three or one target copy are shown in Fig 4 and S3 Fig.

**Fig 4:**
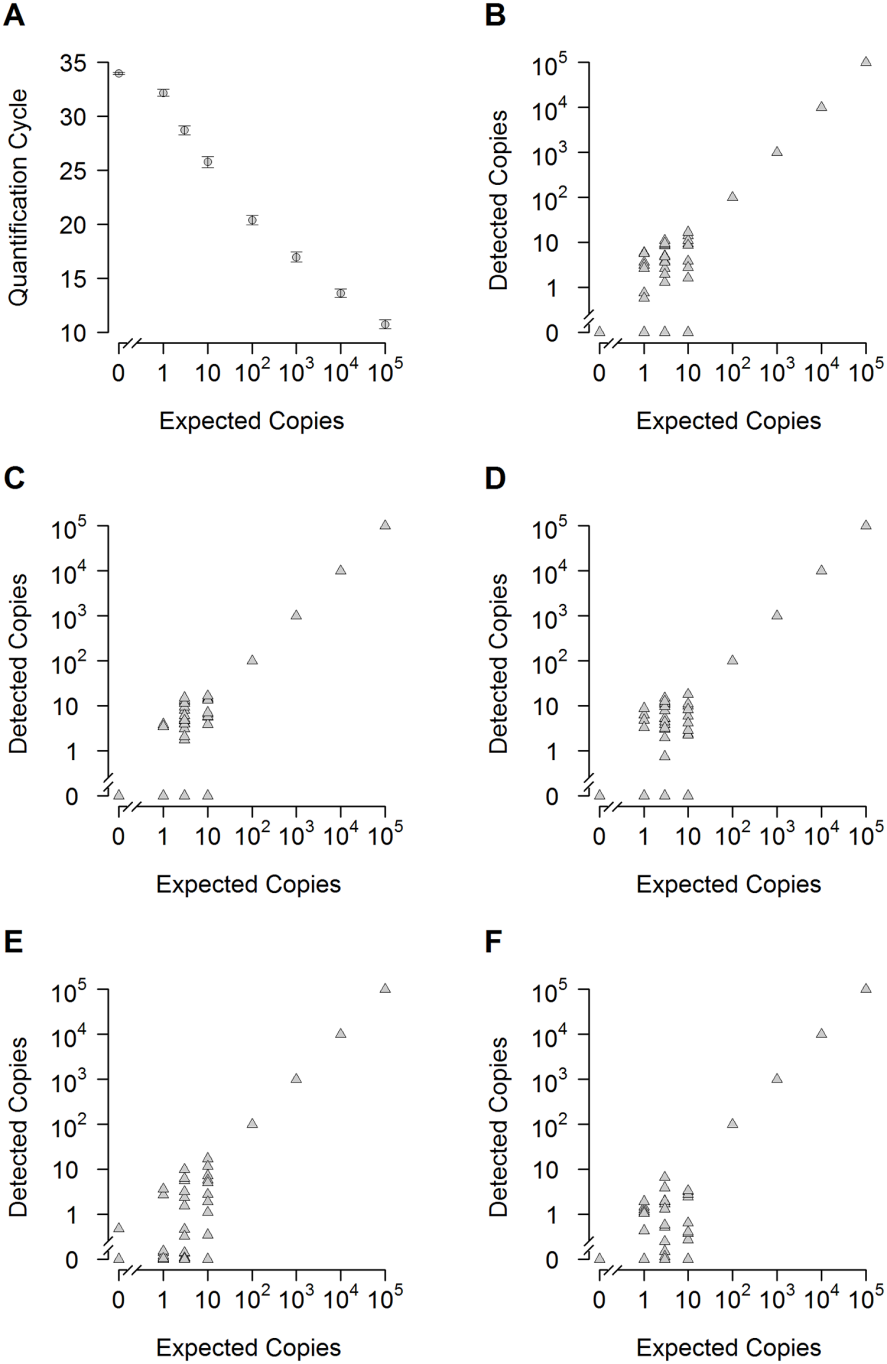
Sensitivity of the *BRAF* V600E assay against a background of 200 000 wild type copies. The sensitivity of detection was analyzed with spike-in experiments. DNA from a melanoma cell line harboring the *BRAF* V600E mutation was spiked against a vast background of DNA from wild type cells (PBMCs). The background DNA equals 2 × 10^5^ copies of wild type BRAF. Number of spiked *BRAF* V600E copies is shown on the x-axis (logarithmic). (A) Quantification cycle of the qPCR (y-axis) is plotted versus the log concentration of mutant DNA per reaction. Circles depict the average Cq value of multiple reactions of five independent experiments (see (B-F)): 0, 1, 3 copies, n = 120; 10 copies, n = 50; 100-10^5^ copies, n = 15). Error bars depict standard error of the mean. (B-F): Scatter plots of five independent spike-in experiments with the number of detected copies shown on the y-axis (logarithmic). Spiked copies are shown on the x-axis (logarithmic). Triangles show the results of single reaction wells (100-10^5^ copies are defined as standards). Number of reactions per qPCR: 0, 1, 3 copies, n = 24; 10 copies, n = 10; 100-10^5^, n = 3. The assay shows reproducibly high sensitivity and specificity. With a single exception (E) all 120 negative controls reactions were negative.

As is typical for ARMS-qPCR, false positive signals due to primer mismatch at high background concentration were visible in our assays (Fig 3, S1 and S2 Figs; orange lines). These false positives derive from primer-elongation despite base-pair mismatch, and define the limit of sensitivity for every assay [46]. In most cases, several Cq values lie between the positive and negative calls (Fig 3). However, at very low copy numbers, especially when comparing a single copy vs. zero, it is possible that the areas of false positive and correctly called signals start to overlap. For these cases, and to generally standardize analysis, a defined algorithm to reliably and reproducibly decide between positive and negative calls is necessary. With two-sided tests, a difference of plus or minus twice the standard error of the mean (±2 × SEM), i.e., a region that covers approximately the 95% confidence interval, is commonly used as such a criterion. Since in our case the test is one-sided, we rather use a difference of ±1.67 × SEM. Therefore, a threshold of the mean of all Cq values of one expected copy minus 1.67 × SEM was calculated for the Cq values of one spiked copy per assay. All calls below this threshold were defined as negative, all above positive. See results section and Fig 4 and S3–S7 Figs for specificity levels of the respective assays.

#### Pairwise comparisons

At very low copy numbers per reaction, i.e. zero (negative control), one, three and ten copies, we tested whether the assays were capable to distinguish between the groups. Linear models with spiked copies and run as factors and the square root of the Cq value were used. For all possible pairwise comparisons of spiked copies within an assay, p-values are reported.

## Results

### Assessment of SNPase-ARMS qPCR assay sensitivity and specificity

To test the flexibility of our system, three assays developed in-house were tested that were specific for the SNVs *BRAF* V600E, *PTEN* T167A and *NRAS* Q61L. Each assay was run at least three times in a defined setup. To analyze the sensitivity of the assays, tumor DNA of cell lines harboring the SNV in question was serially diluted into a constant background of 300 ng or 600 ng of wild type DNA from PBMCs. This background represents 10^5^ and 2 × 10^5^ of wild type copies, respectively. The dilution series of the tumor DNA ranged from 10^5^ copies to one copy per reaction, with a defined 96-well plate layout in all experiments.

#### Dynamic range from 100 000 copies to 100 copies

BRAF mutations are found in around 50% of melanomas, with V600E the most common mutation [51]. Eight qPCR runs were performed with the layout shown in Fig 2. First, to test the overall dynamic range of the *BRAF* V600E assay, dilutions from 10^5^ to 100 copies in a background of 10^5^ copies (three runs) and 2 × 10^5^ copies (five runs) of wild type DNAs, were analyzed. Fig 3 shows the amplification plot of the qPCR for a background of 2 × 10^5^ wild type copies. In the range from 10^5^ to 100 copies the triplicate reactions show very little variation, at an average of ten copies per reaction the variability starts to increase. qPCR runs of the *BRAF* V600E assay showed an average slope of -3.081 (mean efficiency 111.33%) and average Y-intercept of 26.061 for a background of 10^5^ copies and an average slope of -3.270 (mean efficiency 102.55%) and average Y-intercept of 26.927 for a background of 2 × 10^5^ wild type copies, respectively. The coefficient of correlation was always higher than 0.976.

The analysis was repeated for the *PTEN* A167T and *NRAS* Q61L assays analogously. Similar to the *BRAF* V600E assay, in the range from 10^5^ to 100 copies the triplicate reactions show very little variation, at an average of ten copies per reaction the variability starts to increase (S1 and S2 Figs). qPCR runs of the *PTEN* A167T assay showed an average slope of -3.317 (mean efficiency 100.25%) and an average Y-intercept of 28.263 for a background of 10^5^ copies. For a background of 2 × 10^5^ wildtype copies, the average slope was -3.531 (mean efficiency 92.05%) and the average Y-intercept was 28.921. The coefficient of correlation was always higher than 0.986.

qPCR runs of the *NRAS* Q61L assay showed an average slope of -3.240 (mean efficiency 103.89%) and an average Y-intercept of 30.142 for a background of 10^5^ copies and an average slope of -3.461 (mean efficiency 94.56%) and an average Y-intercept of 29.373 for a background of 2 × 10^5^ wildtype copies, respectively. The coefficient of correlation was always higher than 0.985.

#### Specificity and DNA input

A main advantage of qPCR is the possibility to analyze high amounts of DNA in single reactions without further processing. SNPase-ARMS qPCR assays are capable of extreme specificity. We tested two backgrounds of 300 ng or 600 ng wild type DNA, with no adverse effects on assay performance. As is typical for ARMS-qPCR, false positive signals due to primer mismatch at high background concentration were visible (Fig 3). However, they were clearly discernable from ten spiked copies.

#### Detection at low copy numbers

**Fig 5:**
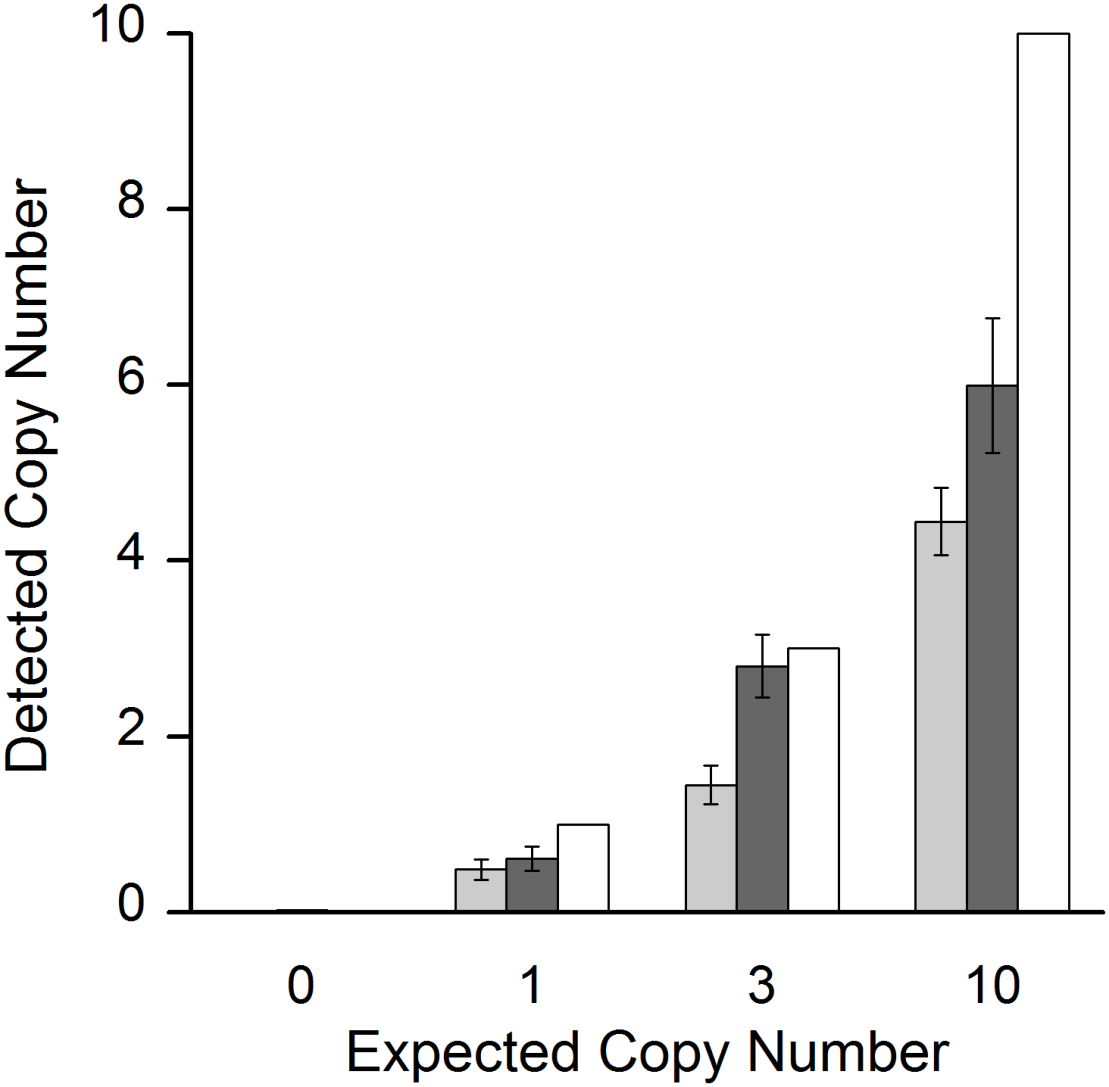
Quantification of low copy numbers of the *BRAF* V600E target mutation. At very low copy numbers, the number of target genes per reaction fluctuates significantly, following Poisson distribution (see also Fig 2). Fig 5 shows the ratio between the average detected copy number (y-axis) v. the expected (= spiked-in) copy number (x-axis and white bars). 0, 1, 3 and 10 *BRAF* V600E copies were spiked against a background of 10^5^ or 2 × 10^5^ copies of wild type DNA (light and dark grey bars respectively). Error bars depict standard error of the mean. The assay somewhat underrates the average copy number at these low concentrations, shown by the difference between grey and white bars. Nevertheless, it correctly detects and differentiates between 0, 1, 3 and 10 spiked copies both against a background of 10^5^ or 2 × 10^5^ wild type copies with unprecedented specificity (p < 0.001 for all pairwise comparisons). Reaction numbers: 2 × 10^5^ copies: 120 for 0, 1 and 3 copies respectively; n = 50 for 10 copies; 10^5^ copies: 72 for 0, 1 and 3 copies respectively; n = 30 for 10 copies.

**Fig 6:**
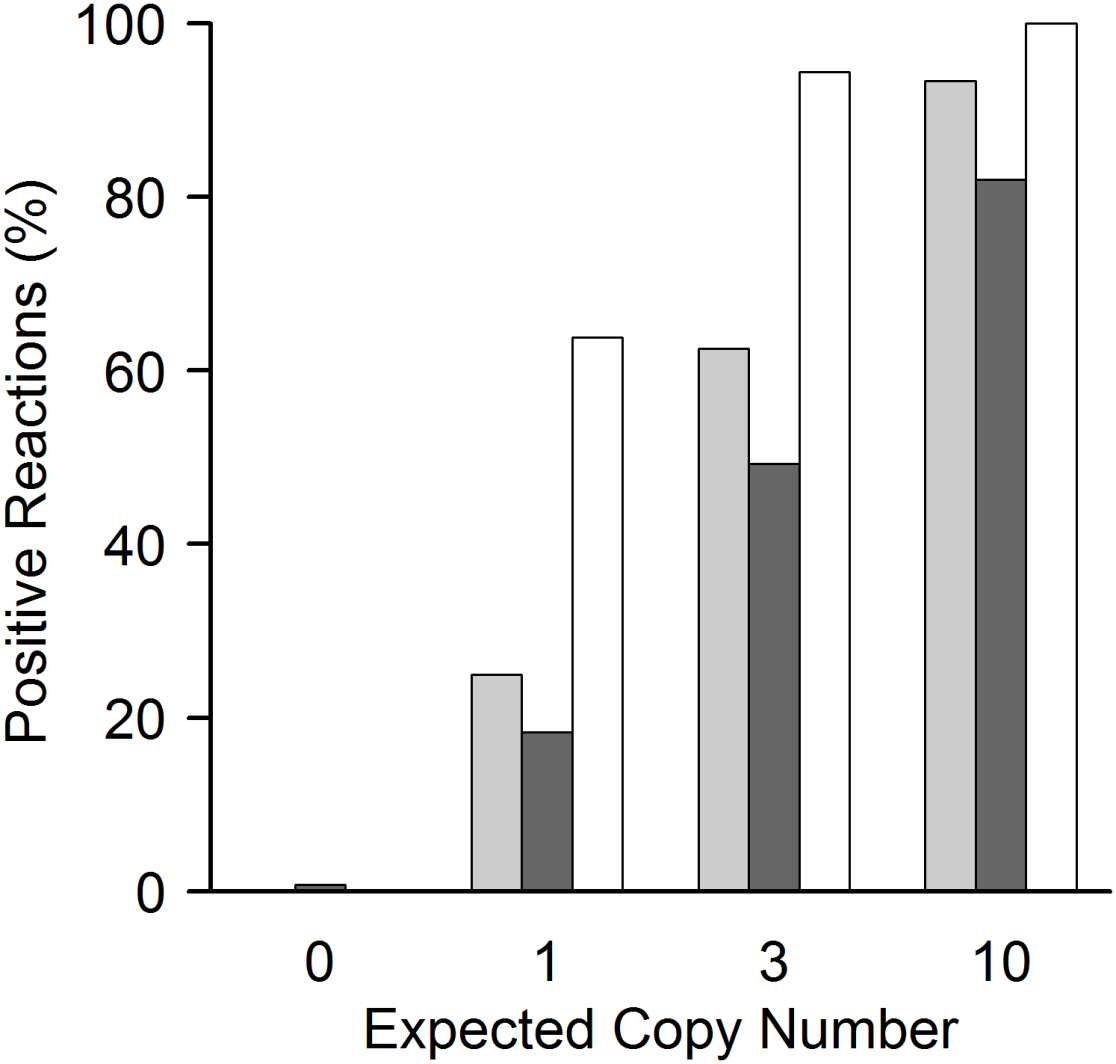
Poisson distribution at low copy numbers of the *BRAF* V600E target mutation. At very low copy numbers, only part of the reaction wells can contain the target gene due to Poisson distribution. Therefore, even under ideal conditions in less than 100% of the reaction wells target DNA can be detected (see also Fig 2). Fig 6 shows the relation between spiked copies (x-axis) and the percentage of positive reactions (y-axis). White bars represent the percentage of reactions that are expected to yield positive signals following ideal Poisson distribution. Light and dark grey columns represent the percentage of reactions that yielded positive signals for *BRAF* V600E detection in a background of 10^5^ and 2 × 10^5^ wild type copies respectively. Reaction numbers: see Fig 5. For details on qPCR plate layout see Fig 2. While at 10 copies per reaction the number of positive wells nearly represents ideal conditions, at 1 and 3 copies the assay detects less than expected positive samples. Reduction of positive calls below 10 starting copies is common in PCR based methods, even without the demanding conditions of mutation detection [49]. Under the extreme sensitivity and specificity constraints tested, the performance of this assay, i.e. correct calling of on average one single mutation per reaction in 2 × 10^5^ wild type DNAs, is unprecedented in qPCR. The reduction of positive calls at very low copy number is the trade-off for extreme specificity. As shown, it can be compensated by the possibility to apply multiple wells per run. The *BRAF* V600E assay correctly detects and differentiates between 0, 1, 3 and 10 spiked copies both against a background of 10^5^ and 2 × 10^5^ wild type copies in our setting (see also Fig 5).

At very low copy numbers, the variance of target DNA in the reaction wells starts to increase. For really low numbers of molecules (i.e., one to ten expected copies), the expected distribution of molecules in a well follows Poisson distribution. In this range, not every reaction well may contain the target sample. To account for this, we increased the number of reaction wells to ten, for ten expected copies; 24 reaction wells for three one and zero expected copies of target DNA, respectively (Fig 2). Results for the *BRAF* V600E assay for a background of 2 × 10^5^ wild type copies of single runs are shown in Fig 4 (see S3 Fig for a background of 10^5^ wild type copies). Detailed analysis of detected copy numbers and the theoretical Poisson distribution are shown in Figs 5 and 6. The *BRAF* V600E assay correctly distinguishes between all groups, i.e. between zero, one, three and ten copies (at p < 0.001 for all pairwise comparisons). However, it somewhat underestimates the total number of copies below ten expected copies, when analysis is extrapolated from the standard curve for 10^5^ to 100 copies (Fig 5). This is also reflected in the reduced number of target-DNA positive wells, when compared to that expected ideally according to the Poisson distribution (Fig 6). Therefore, the underestimation at very low copy numbers is not (solely) due to the reduced efficacy of the assay at these levels, but also on reduced detection. Nevertheless, sensitivity was so high that even a single expected copy of tumor DNA per reaction well could be reproducibly differentiated from samples without spiked copies in a background of 2 × 10^5^ wild type DNA (p < 0.001).

Similar results were obtained for the *PTEN* T167A and *NRAS* Q61L assays. Results of single runs of these two assays are shown in S4–S7 Figs. The analysis of Poisson distribution and detected *PTEN* T167A and *NRAS* Q61L copy numbers are shown in S8–S11 Figs, respectively. The *PTEN* T167A assay is also able to differentiate between no spiked-in copies and one, three or ten tumor copies (at p < 0.001 for all pairwise comparisons) in the presence of 10^5^ copies and 2 × 10^5^ copies of wild type DNA; the *NRAS* Q61L mutation is able to differentiate between no spiked-in copies and three or ten tumor copies (at p < 0.001 for all pairwise comparisons) in the presence of 10^5^ copies and 2 × 10^5^ copies of wild type DNA, but not between zero and one spiked-in copy (p > 0.05 for both 10^5^ and 2 × 10^5^ copies). In the series of dilutions, we observed that at ≥ 3 copies of mutated DNA as a template, the *NRAS* Q61L assay will detect and amplify the mutated gene. As already seen in the *BRAF* V600E assay, the *PTEN* T167A and *NRAS* Q61L assays likewise underestimate the total number of copies below ten (S8 and S9 Figs), which also become apparent in the reduced number of mutant DNA positive reaction wells, in comparison to Poisson distribution (S10 and S11 Figs).

### Clinical application of the *BRAF* V600E assay

To test the clinical applicability of the method, it was applied to a small cohort of melanoma patients. A total of nine melanoma patients were enrolled between July 2013 and September 2014 in the study. Their patients´ characteristics are summarized in Table 3.

**Table 3:** Melanoma Patients' characteristics (TNM classification) and average concentrations of cfDNA and ctDNA in plasma of melanoma patients.

Patient	Stage	TNM class^1^	Therapy	Plasma cfDNA (ng per mL)	*BRAF* V600E (copies)
1	IV	T3bN0M1c	Ipilimumab	287.5	9.48 per 4 ml
2	IV	T4N3M1c	Vemurafenib	193.2	847.78 per 5 ml
3	III A	T2aN1aM0	Interferon	96	0
4	III C	T4bN2cM0	Interferon	176.1	0
5	II C	T4bN0M0	Interferon	110.4	0
6	IV	T1aN0M1c	Vemurafenib	81.2	32.51 per 4 ml
7	I B	T2aN0M0	Interferon	185.5	0.45 per 5 ml
8	IV	T4bN3M1c	Vemurafenib	176	0.48 per 4 ml
9	IV	T3bN1aM1c	Vemurafenib	209.6	20.06 per 4 ml

^1^ According to AJCC classification

As this survey was designed solely to test the clinical applicability of the method, patient blood was drawn during routine care, irrespective of the on-going treatment. Spectrophotometrical quantification of cfDNA revealed a mean concentration of 168.38 ng (interquartile range: 82.8 ng) per mL plasma for melanoma patients. Plasma concentrations of cfDNA were between 81 and 287 ng per mL. *BRAF* V600E copies were found in the range of 0 to over 800 copies per 4 mL and 5 mL plasma, respectively (Table 3). Thirteen analyzed plasma samples from healthy donors yielded a cfDNA concentration of 153.8 ng (interquartile range: 31.8 ng) per mL plasma.

As expected, melanoma patients with advanced disease showed varying levels of *BRAF* V600E, while patients with lower stages of melanoma were negative. The exception was patient 7 whose plasma sample was positive at low levels (analyzed in two independent qPCR runs), despite a low tumor stage (IB). Mean concentration of cfDNA and ctDNA quantified by SNPase-ARMS qPCR of individual melanoma patients are reported in Table 3.

## Discussion

Analysis of cfDNA and ctDNA in patient plasma becomes increasingly important for tumor characterization, treatment options and patient prognosis. While advances in NGS allow detailed genetic analysis of tumor composition, the detection and quantification of tumor-specific mutations in the patient plasma is currently hampered by technical limitations of detection methods. The main problem can be compared to finding “a needle in a haystack”. Most methods are not sensitive enough to detect tumor-specific mutations in the vast background of wild type DNA. In the future, NGS may allow for detecting several, or probably all tumor-specific mutations in the patients’ blood in a single analysis step. But presently NGS is not sensitive enough and technically challenging, even though promising protocols are currently in development [28]. For tumor monitoring, either during treatment or as control of recurrence after excision of the primary tumor, one or several SNVs (to cover possible tumor inhomogeneity) should be sufficient [52, 53]. Currently, a variety of techniques is applied, all of them showing several advantages and disadvantages.

Here we present a novel qPCR protocol that reaches unrivalled sensitivity and specificity. It is based on the successive application of two distinct polymerases. The first polymerase is highly specific for point mutations, and increases the ratio between target and non-target DNA by amplifying target DNA. In a second step, target DNA is quantified with a conventional ARMS qPCR. Detection of the mutant in this step benefits from the improved ratio between target and non-target DNA.

ARMS qPCR with conventional primers and polymerases reaches a SNV detection limit of about 1 SNV in 1000 wild type copies [54]. SNPase polymerase increases this detection limit dramatically owing to its tendency not to elongate primers when a mismatch occurs at the 3′-end. While giving excellent results in discrimination of point mutations, however, in our hands, SNPase as sole polymerase in qPCR did not give satisfying quantifiable results due to its low and varying amplification efficiency. Moreover, SNPase has no 5′ to 3′ exonuclease activity and therefore cannot be combined with hydrolysis probes. However, hydrolysis probes increase specificity due to hybridization to the target sequence [55]. Consecutive application of both polymerases led to both high specificity during the selective preamplification and applicability of hydrolysis probes.

We tested SNPase-ARMS qPCR for its applicability in ctDNA SNV detection. Detection of ctDNA is easy if it is present in several thousand copies per milliliter plasma. But the nature of the problem requires methods to detect only a few copies per milliliter. The reasons why the amount of ctDNA in the plasma varies strongly are currently not fully understood; tumor stage, size, aggressivity, physiological filtering processes of the blood and therapy stage seem to play a role [1, 2]. Due to the variability of SNVs among patients and tumors, applicability of the method to different point mutations is important. To cover these requirements, we tested SNPase-ARMS qPCR independently on three different SNVs, specifically *BRAF* V600E, *PTEN* T167A and *NRAS* Q61L.

Initially, the assays were tested with spike-in experiments, covering gDNA dilutions from 10^5^ copies to one copy of target DNA; zero copies were used as negative controls. As expected, all three assays showed a wide quantitative dynamic range from 10^5^ to ten copies against a wild type DNA background of 10^5^ and 2 × 10^5^ copies. This background of 600 ng wild type gDNA highlights a considerable advantage of qPCR: it is possible to introduce large amounts of DNA into single reactions–a crucial point regarding the sensitivity of low copy number detection [24, 28]. The expected amount of cfDNA per milliliter plasma of cancer patients ranges between 0 and > 1000 ng [3]. The possible input of 600 ng DNA would therefore correspond to 0.5 mL to 60 mL plasma that can be investigated in a single reaction. Thus, great volumes of plasma can easily be processed in a single qPCR run. The low tumor-DNA copy numbers in the plasma make it inevitable to screen several milliliters to ensure detection even under the best conditions, since higher amounts of plasma DNA per reaction generally facilitate detection and quantification. Donation of several milliliters blood is not expected to stress cancer patients.

At very low amounts of ctDNA, it is possible that the target-DNA copy number decreases to below ten copies even when high amounts of cfDNA are analyzed. Detection of low copy numbers not only tests the assay quality to its limits, it also is significantly influenced by a given sample of low copy number, which is expected to follow a Poisson distribution.

We tested the three assays for SNV detection at ten, three, one and zero copies per reaction, again against a background of 10^5^ and 2 × 10^5^ wild type copies. Analysis of a great number of reactions per copy number showed that two of the assays, *BRAF* V600E and *PTEN* T167A, can indeed discriminate between all groups and, most importantly, allow for detection of one expected copy per reaction against a background of 600 ng DNA. The third assay, *NRAS* Q61L, reached a detection limit of 3 copies, i.e. needs on average three molecules of DNA per reaction for reliable detection, again against a background of 600 ng DNA.

It is difficult to achieve a sensitivity of one expected copy of DNA per analysis with qPCR; accordingly such conditions are rarely tested [49]. For allele-specific qPCR detection of two copies per reaction is reported, albeit without checking against the theoretical Poisson distribution [25, 27]. In contrast, digital PCR systems are based on the detection of single target genes in individual micro-compartments. In theory, dPCR is therefore limited only by the number of analyzed compartments. However, also dPCR has been shown to create some false positive results, partly due to replication errors of the polymerase [56]. This limits its detection sensitivity to around 1 in 10 000, with better detection limits reserved for quite complex systems (Table 1). In SNPase-ARMS qPCR the polymerase error rate is low, as only the correct target gets preamplified, in spite of its relatively low copy numbers. While in this case polymerase errors are unlikely, an erroneous amplification start on the non-target template would be possible. The error rate of this processes is surprisingly low, however; we achieved a specificity of up to 1 in 2 × 10^5^ in this study.

Our system thus increases the previously reported detection limit of conventional ARMS qPCR by two orders of magnitude [18, 20, 46], and that of more sophisticated (and demanding) qPCR strategies by at least one order of magnitude [22, 27]. Generally, only digital techniques report sensitivities of up to 1 in 10^5^ copies (Table 1) with only a single report of the detection of 1 in 2 × 10^5^ copies with a complex dPCR method [57]. In summary, SNPase-ARMS qPCR is capable of detecting a single copy of a target, with a broad dynamic range from at least 10^5^ copies to one copy in a background of 2 × 10^5^ non-target copies of DNA–under the condition that enough qPCR reactions are run to account for the sampling distribution of individual copies (Poisson distribution).

So what are the pros and cons of SNPase-ARMS qPCR? Its design is very simple. A common primer-pair designed allele-specifically with the ARMS protocol, i.e. with an additional, intentional mismatch on the third base from the 3′-end, and a dual labeled probe covers almost all possible SNVs. qPCR is nowadays almost universally available. The cfDNA of plasma can be extracted using a commercially available kit, and is ready to use without further processing. The only additional step is the introduction of a second polymerase and the dual labeled hydrolysis probe after an initial preamplification step. The greatest strength of SNPase-ARMS qPCR is its sensitivity enabling the detection of 1 in 2 × 10^5^ copies. The dynamic range is extremely wide, which is generally typical for qPCR, but at the same time, detection of single copies is possible.

Costs per reaction, including all reagents and plastics, are around $0.97 in our setting (the SNPase polymerase with $0.79 per reaction being the most expensive ingredient). This is considerably less than ddPCR ($4, [58]), BEAMing (>$100, [13]) or NGS ($500, [59]). Additionally to these economic advantages, the simultaneous processing of 96 reactions, which can most likely be extended to 384 reactions with an appropriate 384 well block, make the method ideal for routine applications with comparatively high throughput.

A regression of quantification in the range between 10^5^ and ten target copies always resulted in an R^2^ > 0.976, with an average of R^2^ = 0.99. This is slightly below conventional qPCR, probably due to the preamplification step. This slightly higher overall variability is likely caused by the higher variability of initial amplification with the SNPase polymerase.

Compared to dPCR systems, the main advantages of our method are i) the possibility to use more input DNA, which eliminates the need of further processing, and ii) the simple, cost-effective protocol. While it is difficult to compare the methods, detection of single mutant copies among 2 × 10^5^ wild type copies has been reported with dPCR only once before [57]. Compared to conventional ARMS qPCR, our protocol increases the specificity 100-fold and tenfold for more sophisticated qPCR techniques, e.g. the Intplex system or LNA / DNA chimera block qPCR.

What types of SNVs can be detected? Generally all SNVs can be detected for which PCR primers can be designed. In our experience annealing temperatures from 44–62°C are possible. Limitations exist for SNVs that are located in short repeated sequences, as in these areas design of primers may be impossible. Repeated sequences also hinder detection by changing the ratio between mutated and non-mutated DNA, if only one or few parts of the repeated sequences carry an SNV. This applies also to SNVs located in (multiallelic) copy number variations, present both in the genomes of physiological cells [60] and tumors [61]. According to our results, detection is possible even in these cases as long as the ratio between SNV and wild-type carrying DNA in the analyzed DNA (i.e. cfDNA) does not exceed 1 in 2 × 10^5^.

We tested the method on a small cohort of *BRAF* V600E positive melanoma patients of varying tumor stages. Despite the small group size and single (in one case double) sampling of blood per patient, it was possible to distinguish between patients with and without metastases. In two patients, one with metastases and one without, the amount of ctDNA was very low but detectable. A technical replication confirmed the result. In the case of the patient with metastases, response to BRAF inhibitor Vemurafenib may have led to a decline in the amount of mutant cfDNA in the blood. The design of the study did not allow follow-up of the positively tested, but metastasis-free patient. Generally, our clinical results are in accordance with other studies on melanoma patients, and show the reliability of SNPase-ARMS qPCR for clinical applications.

In summary, SNPase-ARMS qPCR is a novel qPCR protocol that allows sensitive and specific detection of SNVs. For routine diagnostics, especially when large amounts of ctDNA need to be screened, we believe it equals or even outperforms sophisticated digital systems due to the possibility of high DNA input, a straightforward protocol and low costs. Moreover it shows that the potential of specially designed polymerases is very likely not exhausted. With further refinement of SNPase polymerase, the preamplification step may be omitted, allowing one-step allele-specific qPCR with the performance of SNPase-ARMS qPCR.

## Supporting Information

S1 Fig Dynamic range of *PTEN* T167A SNPase-ARMS qPCR. S1 Fig shows exemplary qPCR amplification plots of a serial dilution of 10^5^ to ten *PTEN* T167A copies in a background of 2 × 10^5^ (A) and 10^5^ (B) wild type PTEN (following a 15 cycle SNPase preamplification step). The respective target-copy number is indicated in the plot. Delta R (y-axis) is plotted against quantification cycle (x-axis). qPCR threshold level is represented by the grey horizontal line. All reactions containing target DNA (blue) are positive and quantifiable. Negative control samples (orange) show delayed amplification or are negative, albeit less pronounced as in the BRAF assay. No signal amplification was observed in the NTC sample wells. Results of wells containing three and one target copy are shown in S6 and S7 Figs.
(TIF)

S2 Fig Dynamic range of *NRAS* Q61L SNPase-ARMS qPCR. S2 Fig shows exemplary qPCR amplification plots of a serial dilution of 10^5^ to ten *NRAS* Q61L copies in a background of 2 × 10^5^ (A) and 10^5^ (B) wild type NRAS (following a 15 cycle SNPase preamplification step). The respective target-copy number is indicated in the plot. Delta R (y-axis) is plotted against quantification cycle (x-axis). qPCR threshold level is represented by the grey horizontal line. All reactions containing target DNA (blue) are positive and quantifiable with the exception of three negatives at ten copies in (A). Negative control samples (orange) show delayed amplification or are negative, albeit less pronounced as in the BRAF assay. No signal amplification was observed in the NTC sample wells. Results of wells containing three and one target copy are shown in S4 and S5 Figs.
(TIF)

S3 Fig Sensitivity of the *BRAF* V600E assay against a background of 100 000 wild type copies. The sensitivity of detection was analyzed with spike-in experiments. DNA from a melanoma cell line harboring the *BRAF* V600E mutation was spiked against a vast background of DNA from wild type cells (PBMCs). The background DNA equals 10^5^ copies of wild type BRAF. Numbers of spiked *BRAF* V600E copies are shown on the x-axis (logarithmic). (A) Quantification cycle of the qPCR (y-axis) is plotted versus the log concentration of mutant DNA per reaction. Circles depict the average Cq value of multiple reactions (see (B-D)): 0, 1, 3 copies, n = 72; 10 copies, n = 30; 100-10^5^ copies, n = 9; respectively. Error bars depict standard error of the mean. (B-D): Scatter plots of three independent spike-in experiments with the number of detected copies shown on the y-axis (logarithmic). Spiked copies are shown on the x-axis (logarithmic). Triangles show the results of single reaction wells (100-10^5^ copies are defined as standards). Number of reactions per qPCR: 0, 1, 3 copies, n = 24; 10 copies, n = 10; 100-10^5^, n = 3. The assay shows reproducibly high sensitivity and specificity. All 72 negative control reactions were negative.
(TIF)

S4 Fig Sensitivity of the *NRAS* Q61L assay against a background of 100 000 wild type copies. The sensitivity of detection was analyzed with spike-in experiments. DNA from a melanoma cell line harboring the *NRAS* Q61L mutation was spiked against a vast background of DNA from wild type cells (PBMCs). The background DNA equals 10^5^ copies of wild type NRAS. Numbers of spiked *NRAS* Q61L copies are shown on the x-axis (logarithmic). (A) Quantification cycle of the qPCR (y-axis) is plotted versus the log concentration of mutant DNA per reaction. Circles depict the average Cq values of multiple reactions (see (B-D)): 0, 1, 3 copies, n = 72; 10 copies, n = 30; 100-10^5^ copies, n = 9; respectively. Error bars depict standard error of the mean. (B-D): Scatter plots of three independent spike-in experiments with the number of detected copies shown on the y-axis (logarithmic). Spiked copies are shown on the x-axis (logarithmic). Triangles show the results of single reaction wells (100–10^5^ copies are defined as standards). Number of reactions per qPCR: 0, 1, 3 copies, n = 24; 10 copies, n = 10; 100–10^5^, n = 3. The assay shows reproducibly high sensitivity and specificity. All 72 negative control reactions were negative.
(TIF)

S5 Fig Sensitivity of the *NRAS* Q61L assay against a background of 200 000 wild type copies. The sensitivity of detection was analyzed with spike-in experiments. DNA from a melanoma cell line harboring the *NRAS* Q61L mutation was spiked against a vast background of DNA from wild type cells (PBMCs). The background DNA equals 2 × 10^5^ copies of wild type NRAS. Numbers of spiked *NRAS* Q61L copies are shown on the x-axis (logarithmic). (A) Quantification cycle of the qPCR (y-axis) is plotted versus the log concentration of mutant DNA per reaction. Circles depict the average Cq value of multiple reactions (see (B-D)): 0, 1, 3 copies, n = 72; 10 copies, n = 30; 100-10^5^ copies, n = 9; respectively. Error bars depict standard error of the mean. (B-D): Scatter plots of three independent spike-in experiments with the number of detected copies shown on the y-axis (logarithmic). Spiked copies are shown on the x-axis (logarithmic). Triangles show the results of single reaction wells (100–10^5^ copies are defined as standards). Number of reactions per qPCR: 0, 1, 3 copies, n = 24; 10 copies, n = 10; 100–10^5^, n = 3. The assay shows reproducibly high sensitivity and specificity. All 72 negative control reactions were negative.
(TIF)

S6 Fig Sensitivity of the *PTEN* A167T assay against a background of 100 000 wild type copies. The sensitivity of detection was analyzed with spike-in experiments. DNA from a melanoma cell line harboring the *PTEN* A167T mutation was spiked against a vast background of DNA from wild type cells (PBMCs). The background DNA equals 2 × 10^5^ copies of wild type PTEN. Numbers of spiked PTEN A167T copies are shown on the x-axis (logarithmic). (A) Quantification cycle of the qPCR (y-axis) is plotted versus the log concentration of mutant DNA per reaction. Circles depict the average Cq value of multiple reactions (see (B-D)): 0, 1, 3 copies, n = 72; 10 copies, n = 30; 100-10^5^ copies, n = 9; respectively. Error bars depict standard error of the mean. (B-D): Scatter plots of three independent spike-in experiments with the number of detected copies shown on the y-axis (logarithmic). Spiked copies are shown on the x-axis (logarithmic). Triangles show the results of single reaction wells (100-10^5^ copies are defined as standards). Number of reactions per qPCR: 0, 1, 3 copies, n = 24; 10 copies, n = 10; 100-10^5^, n = 3. The assay shows reproducibly high sensitivity and specificity. However, several false-positives were detected in the negative control samples (C-D). Nevertheless, statistical analysis showed that the *PTEN* A167T assay correctly detects and differentiates between 0, 1, 3 and 10 spiked copies.
(TIF)

S7 Fig Sensitivity of the *PTEN* A167T assay against a background of 200 000 wild type copies. The sensitivity of detection was analyzed with spike-in experiments. DNA from a melanoma cell line harboring the *PTEN* A167T mutation was spiked against a vast background of DNA from wild type cells (PBMCs). The background DNA equals 2 × 10^5^ copies of wild type PTEN. Numbers of spiked PTEN A167T copies are shown on the x-axis (logarithmic). (A) Quantification cycle of the qPCR (y-axis) is plotted versus the log concentration of mutant DNA per reaction. Circles depict the average Cq value of multiple reactions (see (B-D)): 0, 1, 3 copies, n = 72; 10 copies, n = 30; 100-10^5^ copies, n = 9; respectively. Error bars depict standard error of the mean. (B-D): Scatter plots of three independent spike-in experiments with the number of detected copies shown on the y-axis (logarithmic). Spiked copies are shown on the x-axis (logarithmic). Triangles show the results of single reaction wells (100–10^5^ copies are defined as standards). Number of reactions per qPCR: 0, 1, 3 copies, n = 24; 10 copies, n = 10; 100-10^5^, n = 3. The assay shows reproducibly high sensitivity and specificity. However, several false-positives were detected in the negative control samples (B, D). Nevertheless, statistical analysis showed that the *PTEN* A167T assay correctly detects and differentiates between 0, 1, 3 and 10 spiked copies.
(TIF)

S8 Fig Quantification of low copy numbers of the *NRAS* Q61L target mutation. At very low copy numbers, the number of target genes per reaction fluctuates significantly, following Poisson distribution (see also Fig 2). S8 Fig shows the ratio between the average detected copy number (y-axis) v the expected (= spiked) copy number (x-axis and white bars). 0, 1, 3 and 10 *NRAS* Q61L copies were spiked against a background of 10^5^ or 2 × 10^5^ copies of wild type DNA (light and dark grey bars respectively). Error bars depict standard error of the mean. The assay somewhat underrates the average copy number at these low concentrations, shown by the difference between grey and white bars. Nevertheless, it correctly detects and differentiates between (0/ 1), 3 and 10 spiked copies both against a background of 10^5^ and 2×10^5^ wild type copies (p < 0.001 for all pairwise comparisons). However, there was no significant difference between 0 and 1 target copy (p > 0.05 for both 10^5^ and 2 × 10^5^ wild type copies), with few positive reaction wells at 1 target copy per well (see S10 Fig). Nevertheless, even with the restriction of three target DNAs per well and a background of 2 × 10^5^ wild type DNAs, the specificity for this assay is still excellent with 1 in 66 000. Reaction numbers: n = 72 for 0, 1 and 3 copies; n = 30 for 10 copies; for both 10^5^ and 2 × 10^5^ background copies. For details on qPCR plate layout see Fig 2.
(TIF)

S9 Fig Quantification of low copy numbers of the *PTEN* A167T target mutation. At very low copy numbers, the number of target genes per reaction fluctuates significantly, following Poisson distribution (see also Fig 2). S9 Fig shows the ratio between the average detected copy number (y-axis) v the expected (= spiked) copy number (x-axis and white bars). 0, 1, 3 and 10 *PTEN* A167T copies were spiked against a background of 10^5^ or 2 × 10^5^ copies of wild type DNA (light and dark grey bars respectively). Error bars depict standard error of the mean. The assay somewhat underrates the average copy number at these low concentrations, shown by the difference between grey and white bars. Nevertheless, it correctly detects and differentiates between 0, 1, 3 and 10 spiked copies both against a background of 10^5^ or 2 × 10^5^ wild type copies with unprecedented specificity (p < 0.001 for all pairwise comparisons). Reaction numbers: n = 72 for 0, 1 and 3 copies; n = 30 for 10 copies; for both 10^5^ and 2 × 10^5^ background copies. For details on qPCR plate layout see Fig 2.
(TIF)

S10 Fig Poisson distribution at low copy numbers of the *NRAS* Q61L target mutation. At very low copy numbers, only part of the reaction wells can contain the target gene due to Poisson distribution. Therefore, even under ideal conditions in less than 100% of the reaction wells target DNA can be detected (see also Fig 2). S10 Fig shows the relation between spiked copies (x-axis) and the percentage of positive reactions (y-axis). White bars represent the percentage of reactions that are expected to yield positive signals following ideal Poisson distribution. Light and dark grey columns represent the percentage of reactions that yielded positive signals for *NRAS* Q61L detection in a background of 10^5^ and 2 × 10^5^ wild type copies respectively. Reaction numbers: see S8 Fig for details on qPCR plate layout see Fig 2. While at 10 copies per reaction the number of positive wells nearly represents ideal conditions, at 1 and 3 copies the assay detects less than expected positive samples. Reduction of positive calls below 10 starting copies is common in PCR based methods, even without the demanding conditions of mutation detection [49]. The reduction of positive calls at very low copy number is the trade-off for extreme specificity. As shown, it can be compensated by the possibility apply multiple wells per run. The *NRAS* Q61L assay correctly detects and differentiates between (0/ 1), 3 and 10 spiked copies both against a background of 10^5^ and 2 × 10^5^ wild type copies in our setting (p < 0.001 for all pairwise comparisons), but not between 0 and 1 copy (p > 0.05 for both 10^5^ and 2 × 10^5^ wild type copies) (S8 Fig). Nevertheless, it shows excellent specificity, with the restriction that three copies of target samples are needed for successful detection.
(TIF)

S11 Fig Poisson distribution at low copy numbers of the *PTEN* A167T target mutation. At very low copy numbers, only part of the reaction wells can contain the target gene due to Poisson distribution. Therefore, even under ideal conditions in less than 100% of the reaction wells target DNA can be detected (see also Fig 2). S11 Fig shows the relation between spiked copies (x-axis) and the percentage of positive reactions (y-axis). White bars represent the percentage of reactions that are expected to yield positive signals following ideal Poisson distribution. Light and dark grey columns represent the percentage of reactions that yielded positive signals for *PTEN* A167T detection in a background of 10^5^ and 2 × 10^5^ wild type copies respectively. Reaction numbers: see S9 Fig for details on qPCR plate layout see Fig 2. While at 10 copies per reaction the number of positive wells nearly represents ideal conditions, at 1 and 3 copies the assay detects less than expected positive samples. Reduction of positive calls below 10 starting copies is common in PCR based methods, even without the demanding conditions of mutation detection [49]. Under the extreme sensitivity and specificity constraints tested, the performance of this assay, i.e. correct calling of on average one single mutation per reaction in 2 × 10^5^ wild type DNAs, is unprecedented in qPCR. The reduction of positive calls at very low copy number is the trade-off for extreme specificity. As shown, it can be compensated by the possibility apply multiple wells per run. The *PTEN* A167T assay correctly detects and differentiates between 0, 1, 3 and 10 spiked copies both against a background of 10^5^ and 2 × 10^5^ wild type copies in our setting (S9 Fig).
(TIF)
