# Supplementary figures and images for: SNPase-ARMS qPCR: Ultrasensitive Mutation-Based Detection of Cell-Free Tumor DNA in Melanoma Patients
Source: PLoS One. 2015 Nov 12;10(11):e0142273. doi: 10.1371/journal.pone.0142273 (PMC4642939; doi:10.1371/journal.pone.0142273)

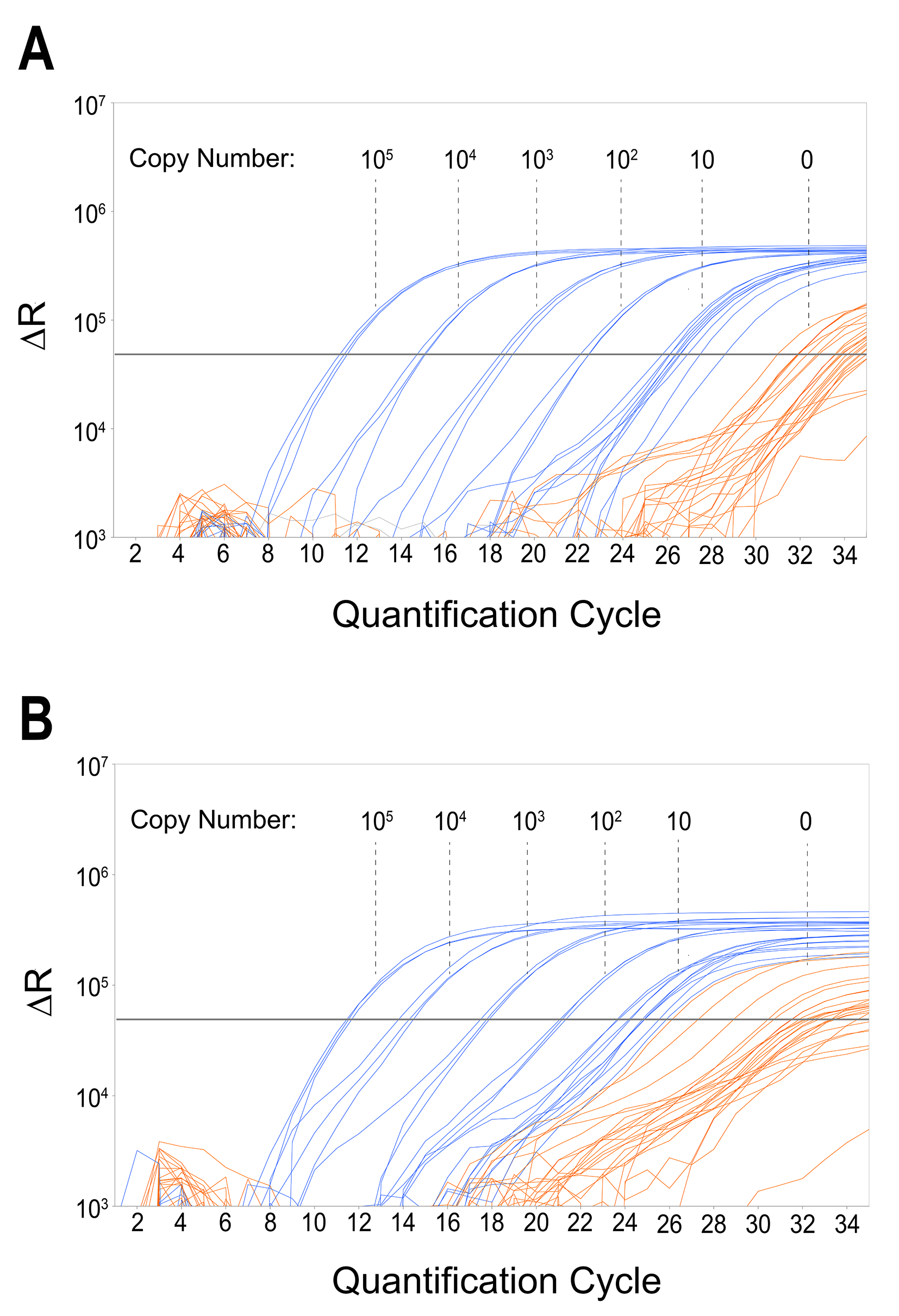

Supplement: S1 Fig — shows exemplary qPCR amplification plots of a serial dilution of 105 to ten PTEN T167A copies in a background of 2 × 10^5^ (A) and 105 (B) wild type PTEN (following a 15 cycle SNPase preamplification step). The respective target-copy number is indicated in the plot. Delta R (y-axis) is plotted against quantification cycle (x-axis). qPCR threshold level is represented by the grey horizontal line. All reactions containing target DNA (blue) are positive and quantifiable. Negative control samples (orange) show delayed amplification or are negative, albeit less pronounced as in the BRAF assay. No signal amplification was observed in the NTC sample wells. Results of wells containing three and one target copy are shown in S6 and S7 Figs. (TIF) [file pone.0142273.s001.tif]

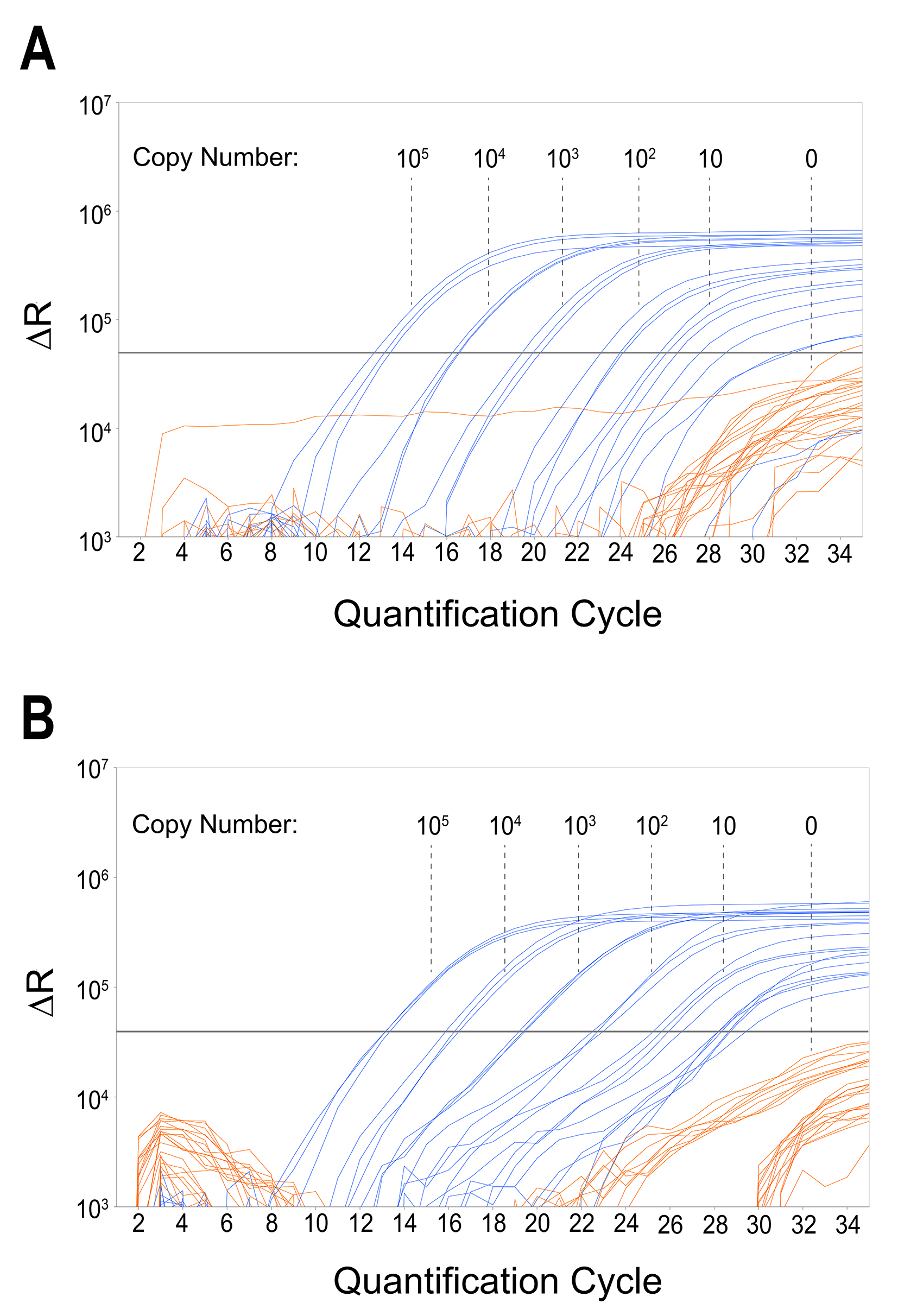

Supplement: S2 Fig — shows exemplary qPCR amplification plots of a serial dilution of 105 to ten NRAS Q61L copies in a background of 2 × 10^5^ (A) and 105 (B) wild type NRAS (following a 15 cycle SNPase preamplification step). The respective target-copy number is indicated in the plot. Delta R (y-axis) is plotted against quantification cycle (x-axis). qPCR threshold level is represented by the grey horizontal line. All reactions containing target DNA (blue) are positive and quantifiable with the exception of three negatives at ten copies in (A). Negative control samples (orange) show delayed amplification or are negative, albeit less pronounced as in the BRAF assay. No signal amplification was observed in the NTC sample wells. Results of wells containing three and one target copy are shown in S4 and S5 Figs. (TIF) [file pone.0142273.s002.tif]

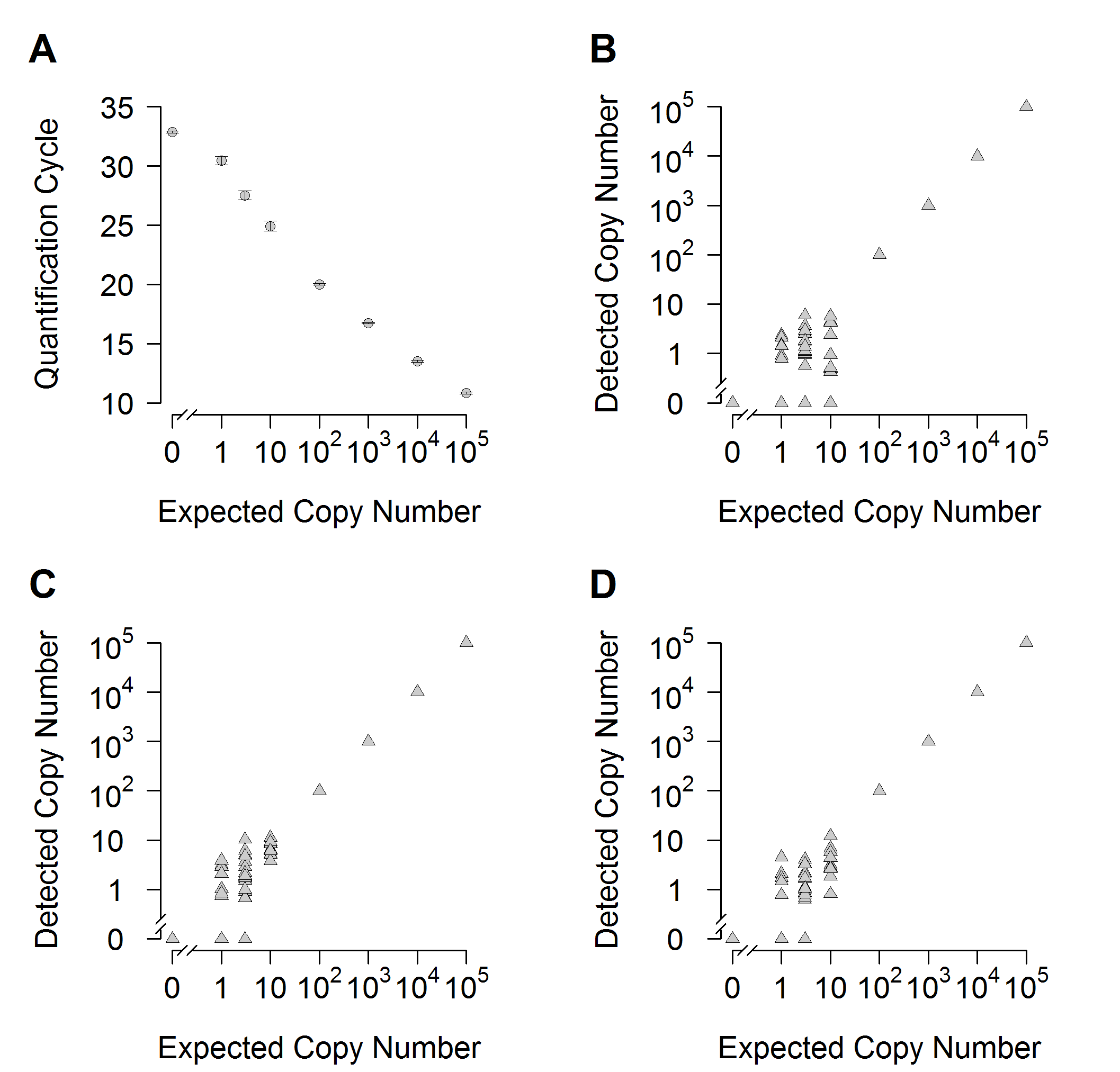

Supplement: S3 Fig — The sensitivity of detection was analyzed with spike-in experiments. DNA from a melanoma cell line harboring the BRAF V600E mutation was spiked against a vast background of DNA from wild type cells (PBMCs). The background DNA equals 105 copies of wild type BRAF. Numbers of spiked BRAF V600E copies are shown on the x-axis (logarithmic). (A) Quantification cycle of the qPCR (y-axis) is plotted versus the log concentration of mutant DNA per reaction. Circles depict the average Cq value of multiple reactions (see (B-D)): 0, 1, 3 copies, n = 72; 10 copies, n = 30; 100-105 copies, n = 9; respectively. Error bars depict standard error of the mean. (B-D): Scatter plots of three independent spike-in experiments with the number of detected copies shown on the y-axis (logarithmic). Spiked copies are shown on the x-axis (logarithmic). Triangles show the results of single reaction wells (100-105 copies are defined as standards). Number of reactions per qPCR: 0, 1, 3 copies, n = 24; 10 copies, n = 10; 100-105, n = 3. The assay shows reproducibly high sensitivity and specificity. All 72 negative control reactions were negative. (TIF) [file pone.0142273.s003.tif]

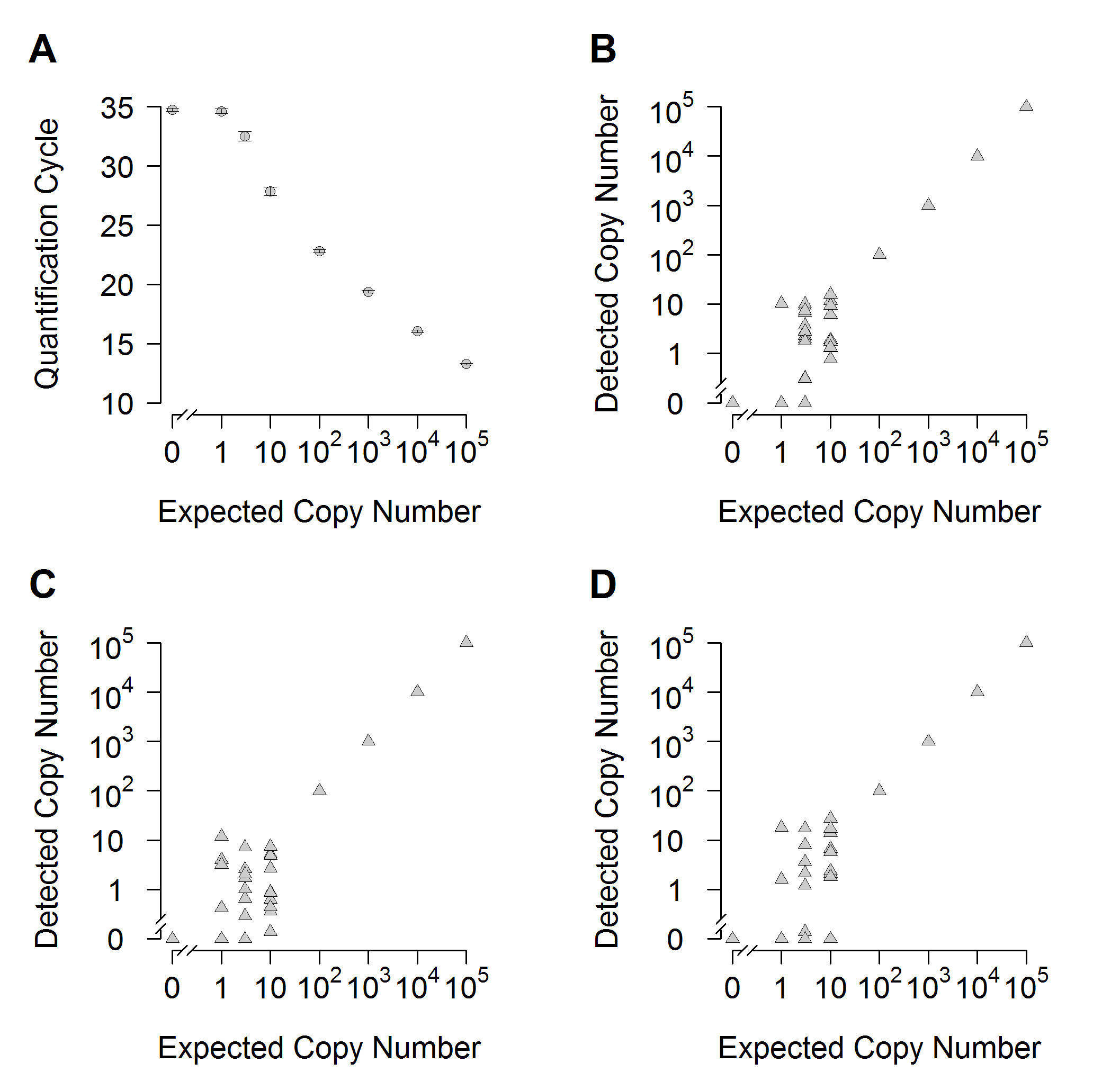

Supplement: S4 Fig — The sensitivity of detection was analyzed with spike-in experiments. DNA from a melanoma cell line harboring the NRAS Q61L mutation was spiked against a vast background of DNA from wild type cells (PBMCs). The background DNA equals 105 copies of wild type NRAS. Numbers of spiked NRAS Q61L copies are shown on the x-axis (logarithmic). (A) Quantification cycle of the qPCR (y-axis) is plotted versus the log concentration of mutant DNA per reaction. Circles depict the average Cq values of multiple reactions (see (B-D)): 0, 1, 3 copies, n = 72; 10 copies, n = 30; 100-105 copies, n = 9; respectively. Error bars depict standard error of the mean. (B-D): Scatter plots of three independent spike-in experiments with the number of detected copies shown on the y-axis (logarithmic). Spiked copies are shown on the x-axis (logarithmic). Triangles show the results of single reaction wells (100–105 copies are defined as standards). Number of reactions per qPCR: 0, 1, 3 copies, n = 24; 10 copies, n = 10; 100–105, n = 3. The assay shows reproducibly high sensitivity and specificity. All 72 negative control reactions were negative. (TIF) [file pone.0142273.s004.tif]

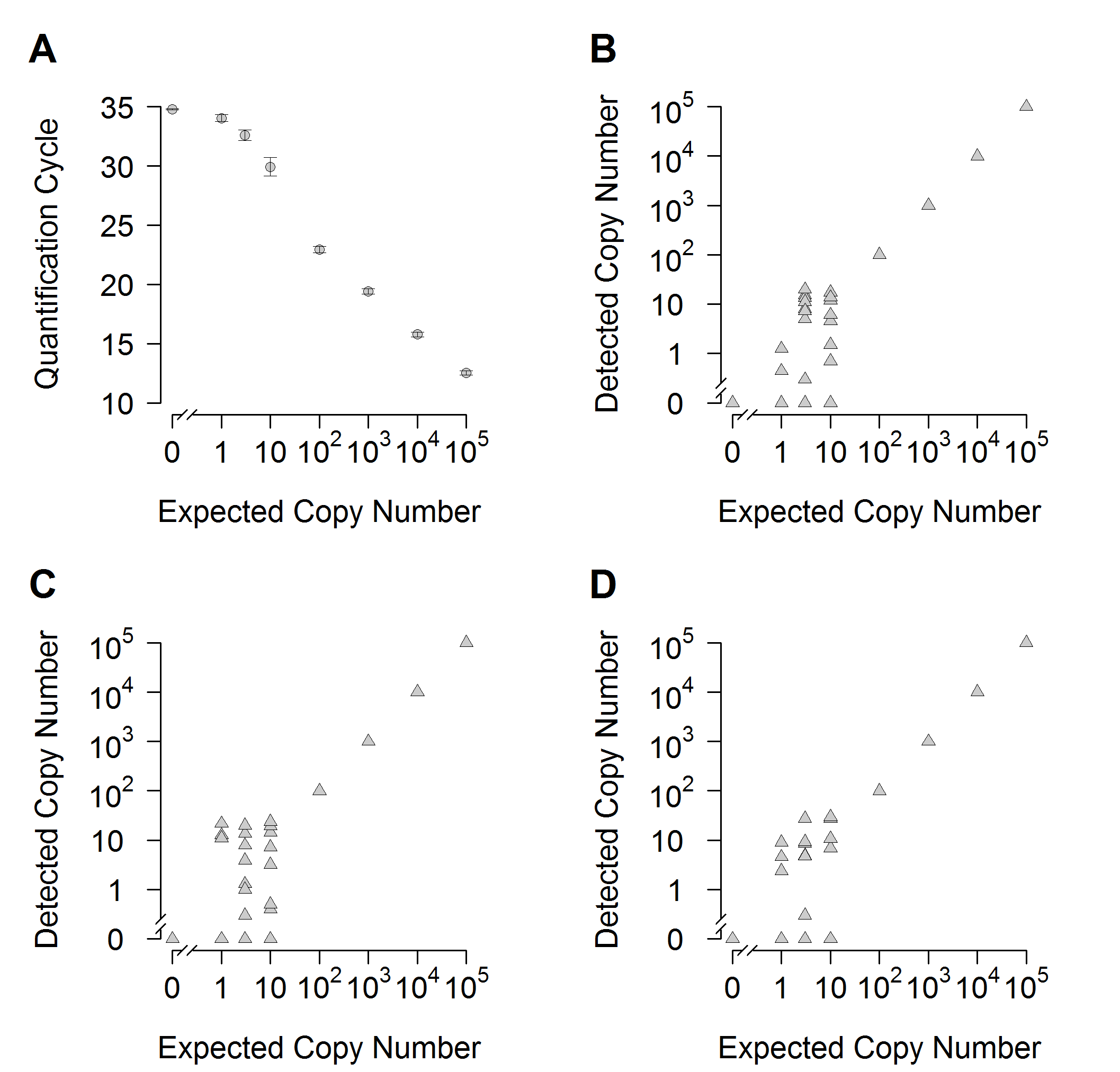

Supplement: S5 Fig — The sensitivity of detection was analyzed with spike-in experiments. DNA from a melanoma cell line harboring the NRAS Q61L mutation was spiked against a vast background of DNA from wild type cells (PBMCs). The background DNA equals 2 × 10^5^ copies of wild type NRAS. Numbers of spiked NRAS Q61L copies are shown on the x-axis (logarithmic). (A) Quantification cycle of the qPCR (y-axis) is plotted versus the log concentration of mutant DNA per reaction. Circles depict the average Cq value of multiple reactions (see (B-D)): 0, 1, 3 copies, n = 72; 10 copies, n = 30; 100-105 copies, n = 9; respectively. Error bars depict standard error of the mean. (B-D): Scatter plots of three independent spike-in experiments with the number of detected copies shown on the y-axis (logarithmic). Spiked copies are shown on the x-axis (logarithmic). Triangles show the results of single reaction wells (100–105 copies are defined as standards). Number of reactions per qPCR: 0, 1, 3 copies, n = 24; 10 copies, n = 10; 100–105, n = 3. The assay shows reproducibly high sensitivity and specificity. All 72 negative control reactions were negative. (TIF) [file pone.0142273.s005.tif]

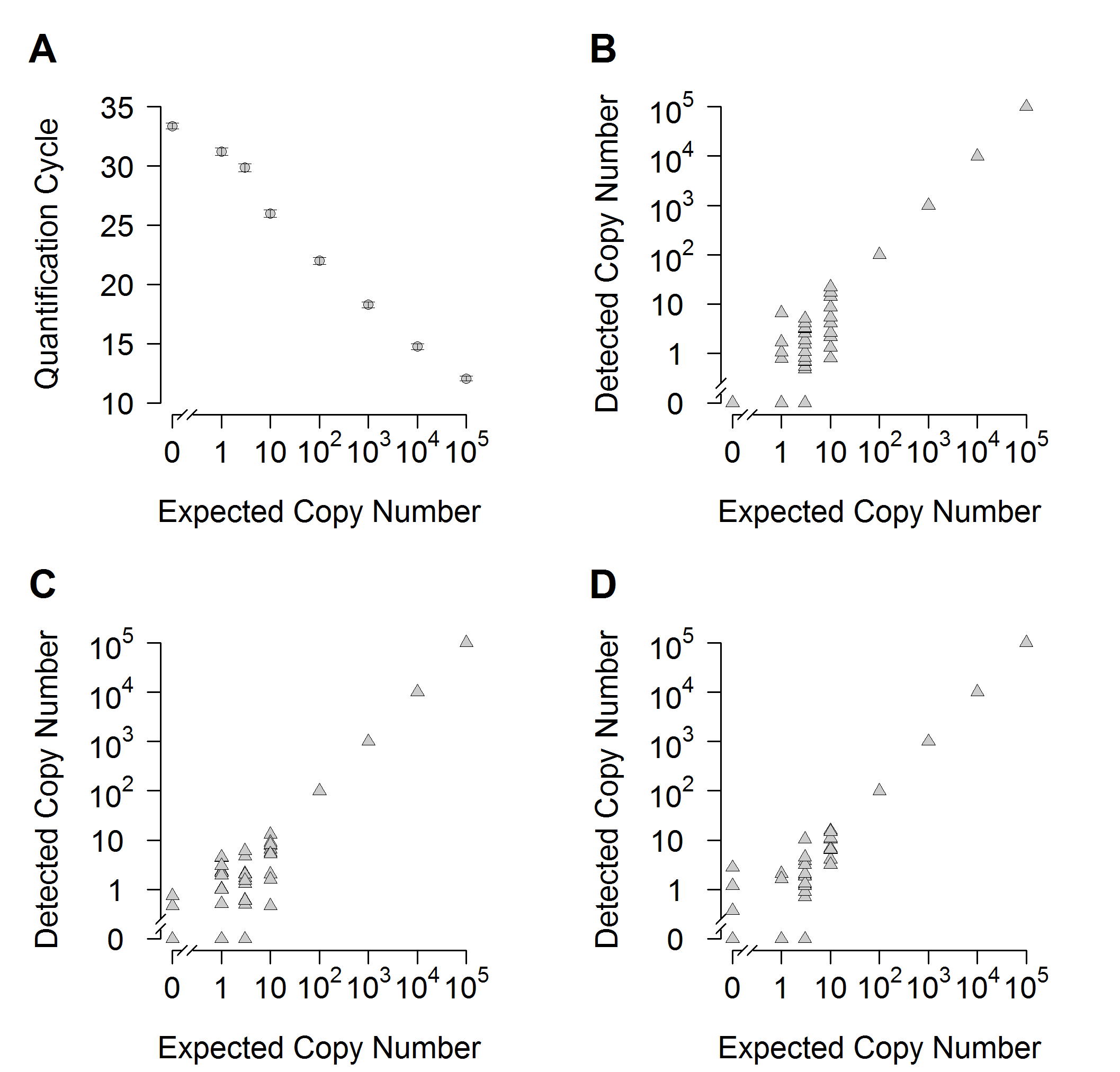

Supplement: S6 Fig — The sensitivity of detection was analyzed with spike-in experiments. DNA from a melanoma cell line harboring the PTEN A167T mutation was spiked against a vast background of DNA from wild type cells (PBMCs). The background DNA equals 2 × 10^5^ copies of wild type PTEN. Numbers of spiked PTEN A167T copies are shown on the x-axis (logarithmic). (A) Quantification cycle of the qPCR (y-axis) is plotted versus the log concentration of mutant DNA per reaction. Circles depict the average Cq value of multiple reactions (see (B-D)): 0, 1, 3 copies, n = 72; 10 copies, n = 30; 100-105 copies, n = 9; respectively. Error bars depict standard error of the mean. (B-D): Scatter plots of three independent spike-in experiments with the number of detected copies shown on the y-axis (logarithmic). Spiked copies are shown on the x-axis (logarithmic). Triangles show the results of single reaction wells (100-105 copies are defined as standards). Number of reactions per qPCR: 0, 1, 3 copies, n = 24; 10 copies, n = 10; 100-105, n = 3. The assay shows reproducibly high sensitivity and specificity. However, several false-positives were detected in the negative control samples (C-D). Nevertheless, statistical analysis showed that the PTEN A167T assay correctly detects and differentiates between 0, 1, 3 and 10 spiked copies. (TIF) [file pone.0142273.s006.tif]

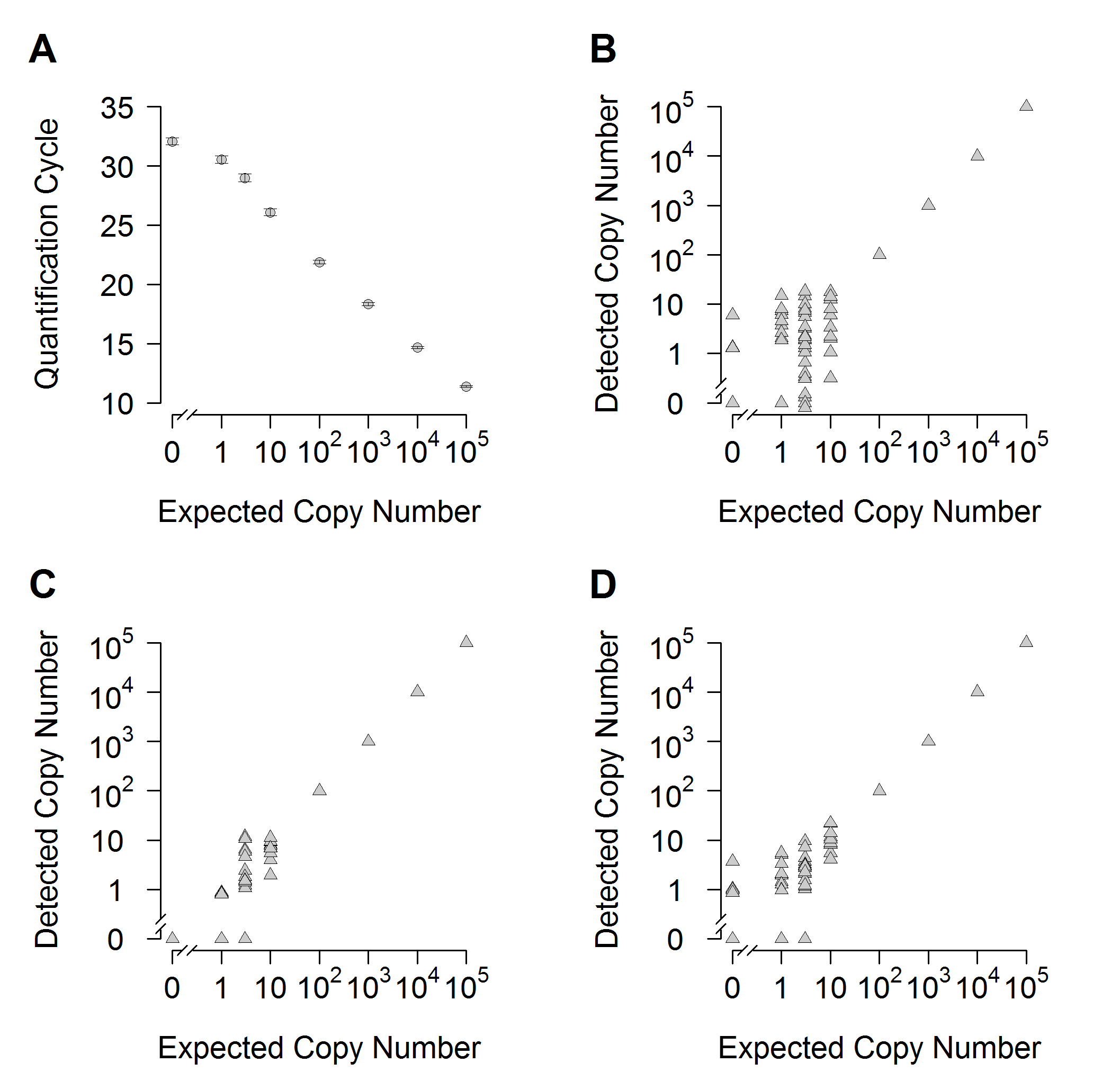

Supplement: S7 Fig — The sensitivity of detection was analyzed with spike-in experiments. DNA from a melanoma cell line harboring the PTEN A167T mutation was spiked against a vast background of DNA from wild type cells (PBMCs). The background DNA equals 2 × 10^5^ copies of wild type PTEN. Numbers of spiked PTEN A167T copies are shown on the x-axis (logarithmic). (A) Quantification cycle of the qPCR (y-axis) is plotted versus the log concentration of mutant DNA per reaction. Circles depict the average Cq value of multiple reactions (see (B-D)): 0, 1, 3 copies, n = 72; 10 copies, n = 30; 100-105 copies, n = 9; respectively. Error bars depict standard error of the mean. (B-D): Scatter plots of three independent spike-in experiments with the number of detected copies shown on the y-axis (logarithmic). Spiked copies are shown on the x-axis (logarithmic). Triangles show the results of single reaction wells (100–105 copies are defined as standards). Number of reactions per qPCR: 0, 1, 3 copies, n = 24; 10 copies, n = 10; 100-105, n = 3. The assay shows reproducibly high sensitivity and specificity. However, several false-positives were detected in the negative control samples (B, D). Nevertheless, statistical analysis showed that the PTEN A167T assay correctly detects and differentiates between 0, 1, 3 and 10 spiked copies. (TIF) [file pone.0142273.s007.tif]

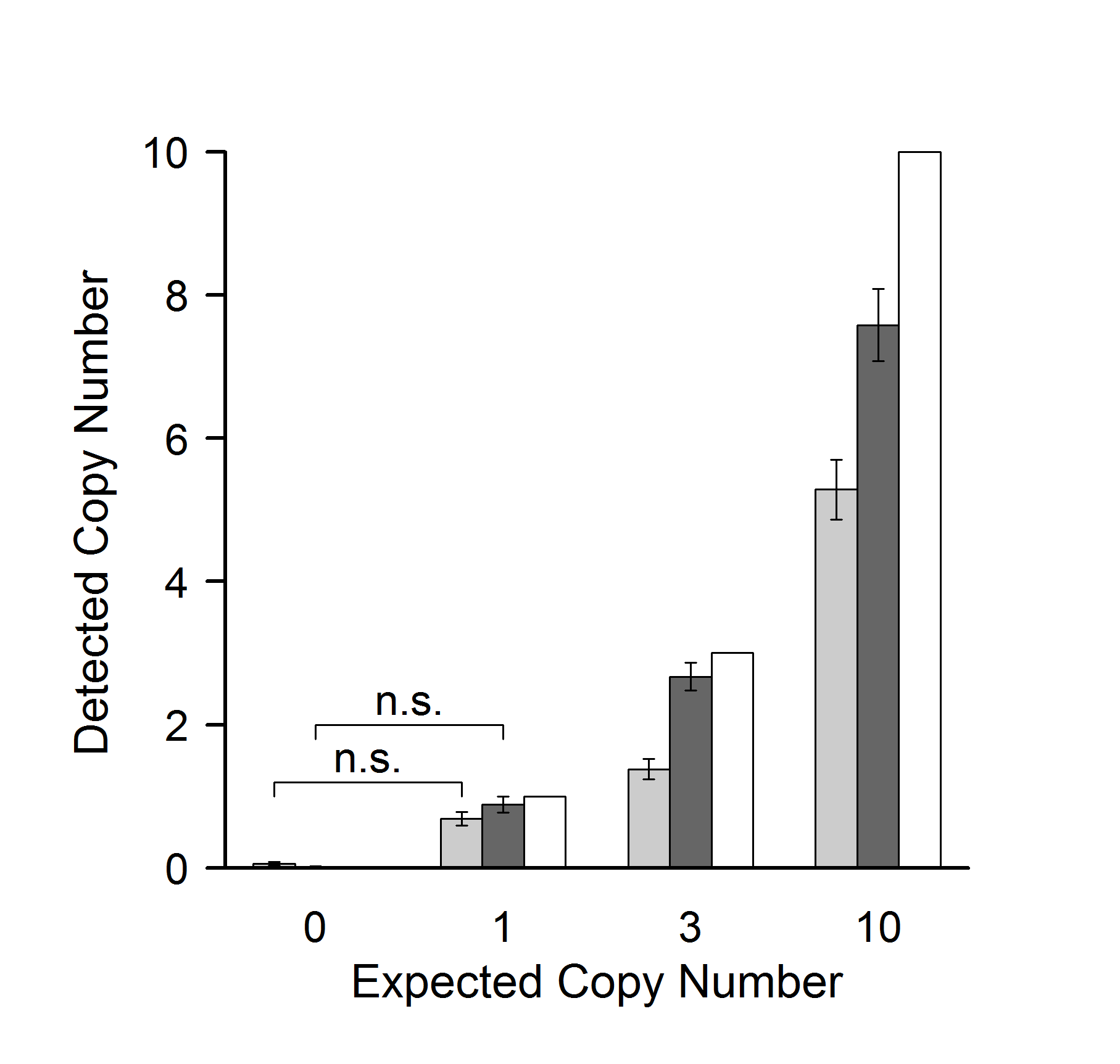

Supplement: S8 Fig — At very low copy numbers, the number of target genes per reaction fluctuates significantly, following Poisson distribution (see also Fig 2). S8 Fig shows the ratio between the average detected copy number (y-axis) v the expected (= spiked) copy number (x-axis and white bars). 0, 1, 3 and 10 NRAS Q61L copies were spiked against a background of 105 or 2 × 10^5^ copies of wild type DNA (light and dark grey bars respectively). Error bars depict standard error of the mean. The assay somewhat underrates the average copy number at these low concentrations, shown by the difference between grey and white bars. Nevertheless, it correctly detects and differentiates between (0/ 1), 3 and 10 spiked copies both against a background of 105 and 2×10^5^ wild type copies (p < 0.001 for all pairwise comparisons). However, there was no significant difference between 0 and 1 target copy (p > 0.05 for both 105 and 2 × 10^5^ wild type copies), with few positive reaction wells at 1 target copy per well (see S10 Fig). Nevertheless, even with the restriction of three target DNAs per well and a background of 2 × 10^5^ wild type DNAs, the specificity for this assay is still excellent with 1 in 66 000. Reaction numbers: n = 72 for 0, 1 and 3 copies; n = 30 for 10 copies; for both 105 and 2 × 10^5^ background copies. For details on qPCR plate layout see Fig 2. (TIF) [file pone.0142273.s008.tif]

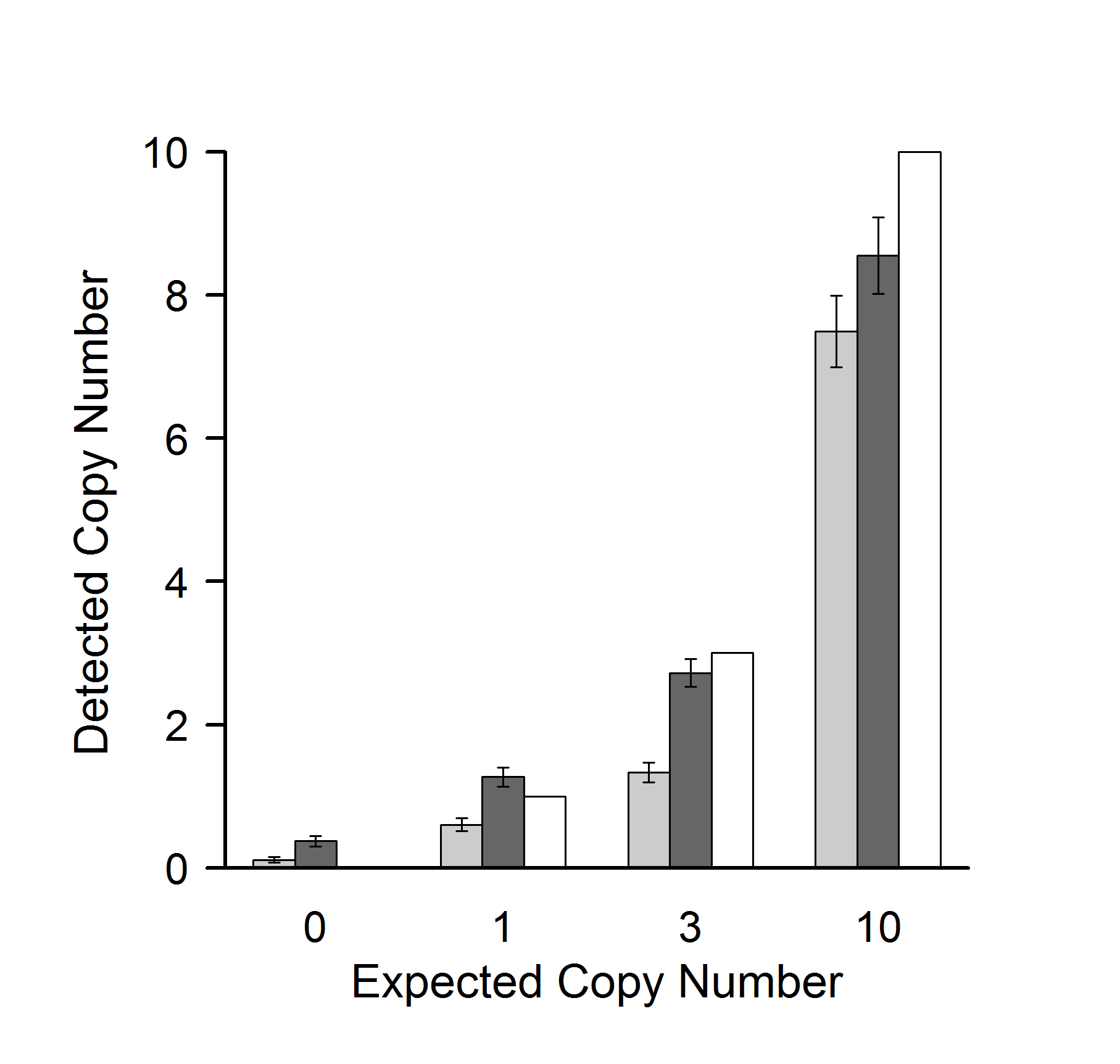

Supplement: S9 Fig — At very low copy numbers, the number of target genes per reaction fluctuates significantly, following Poisson distribution (see also Fig 2). S9 Fig shows the ratio between the average detected copy number (y-axis) v the expected (= spiked) copy number (x-axis and white bars). 0, 1, 3 and 10 PTEN A167T copies were spiked against a background of 105 or 2 × 10^5^ copies of wild type DNA (light and dark grey bars respectively). Error bars depict standard error of the mean. The assay somewhat underrates the average copy number at these low concentrations, shown by the difference between grey and white bars. Nevertheless, it correctly detects and differentiates between 0, 1, 3 and 10 spiked copies both against a background of 105 or 2 × 10^5^ wild type copies with unprecedented specificity (p < 0.001 for all pairwise comparisons). Reaction numbers: n = 72 for 0, 1 and 3 copies; n = 30 for 10 copies; for both 105 and 2 × 10^5^ background copies. For details on qPCR plate layout see Fig 2. (TIF) [file pone.0142273.s009.tif]

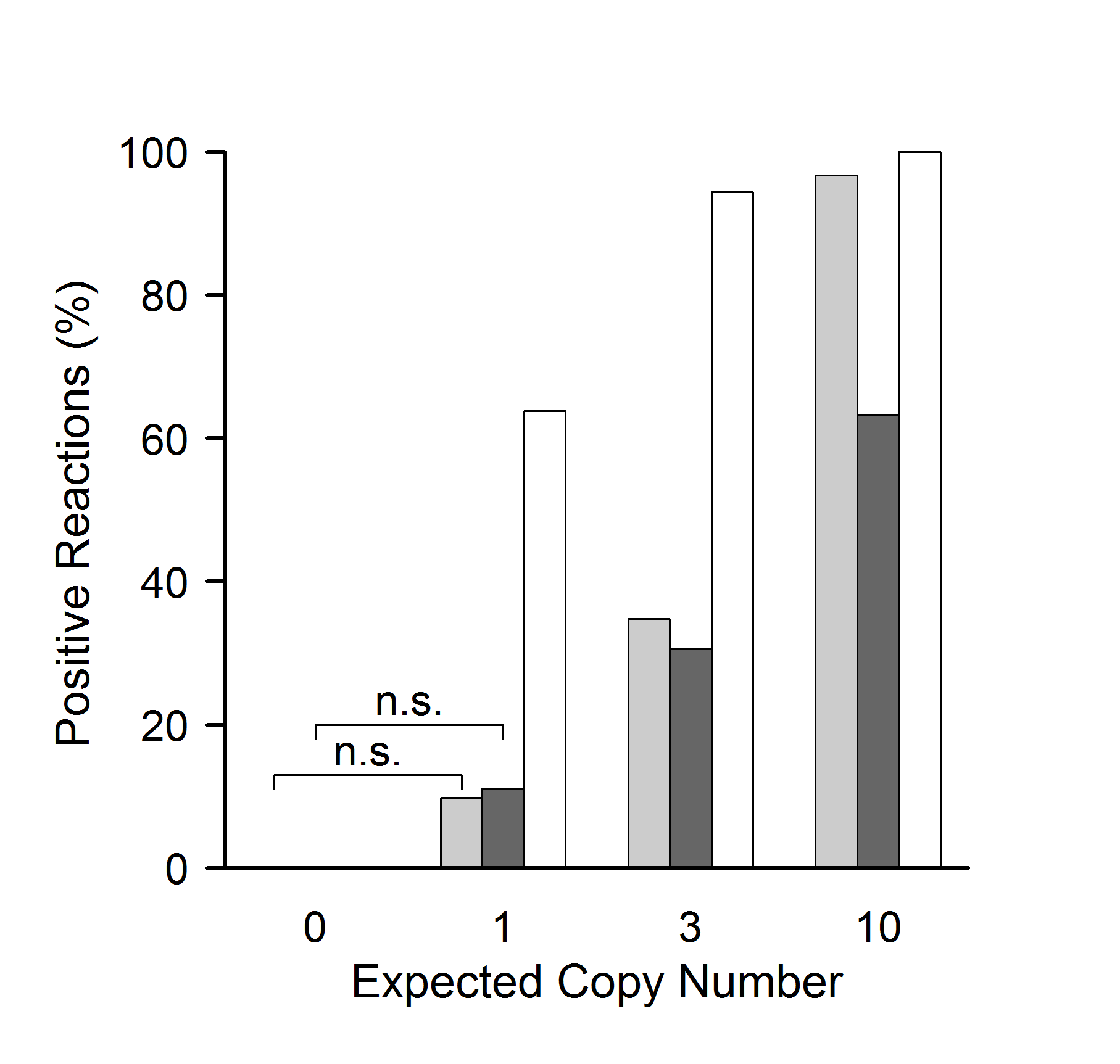

Supplement: S10 Fig — At very low copy numbers, only part of the reaction wells can contain the target gene due to Poisson distribution. Therefore, even under ideal conditions in less than 100% of the reaction wells target DNA can be detected (see also Fig 2). S10 Fig shows the relation between spiked copies (x-axis) and the percentage of positive reactions (y-axis). White bars represent the percentage of reactions that are expected to yield positive signals following ideal Poisson distribution. Light and dark grey columns represent the percentage of reactions that yielded positive signals for NRAS Q61L detection in a background of 105 and 2 × 10^5^ wild type copies respectively. Reaction numbers: see S8 Fig for details on qPCR plate layout see Fig 2. While at 10 copies per reaction the number of positive wells nearly represents ideal conditions, at 1 and 3 copies the assay detects less than expected positive samples. Reduction of positive calls below 10 starting copies is common in PCR based methods, even without the demanding conditions of mutation detection [49]. The reduction of positive calls at very low copy number is the trade-off for extreme specificity. As shown, it can be compensated by the possibility apply multiple wells per run. The NRAS Q61L assay correctly detects and differentiates between (0/ 1), 3 and 10 spiked copies both against a background of 105 and 2 × 10^5^ wild type copies in our setting (p < 0.001 for all pairwise comparisons), but not between 0 and 1 copy (p > 0.05 for both 105 and 2 × 10^5^ wild type copies) (S8 Fig). Nevertheless, it shows excellent specificity, with the restriction that three copies of target samples are needed for successful detection. (TIF) [file pone.0142273.s010.tif]

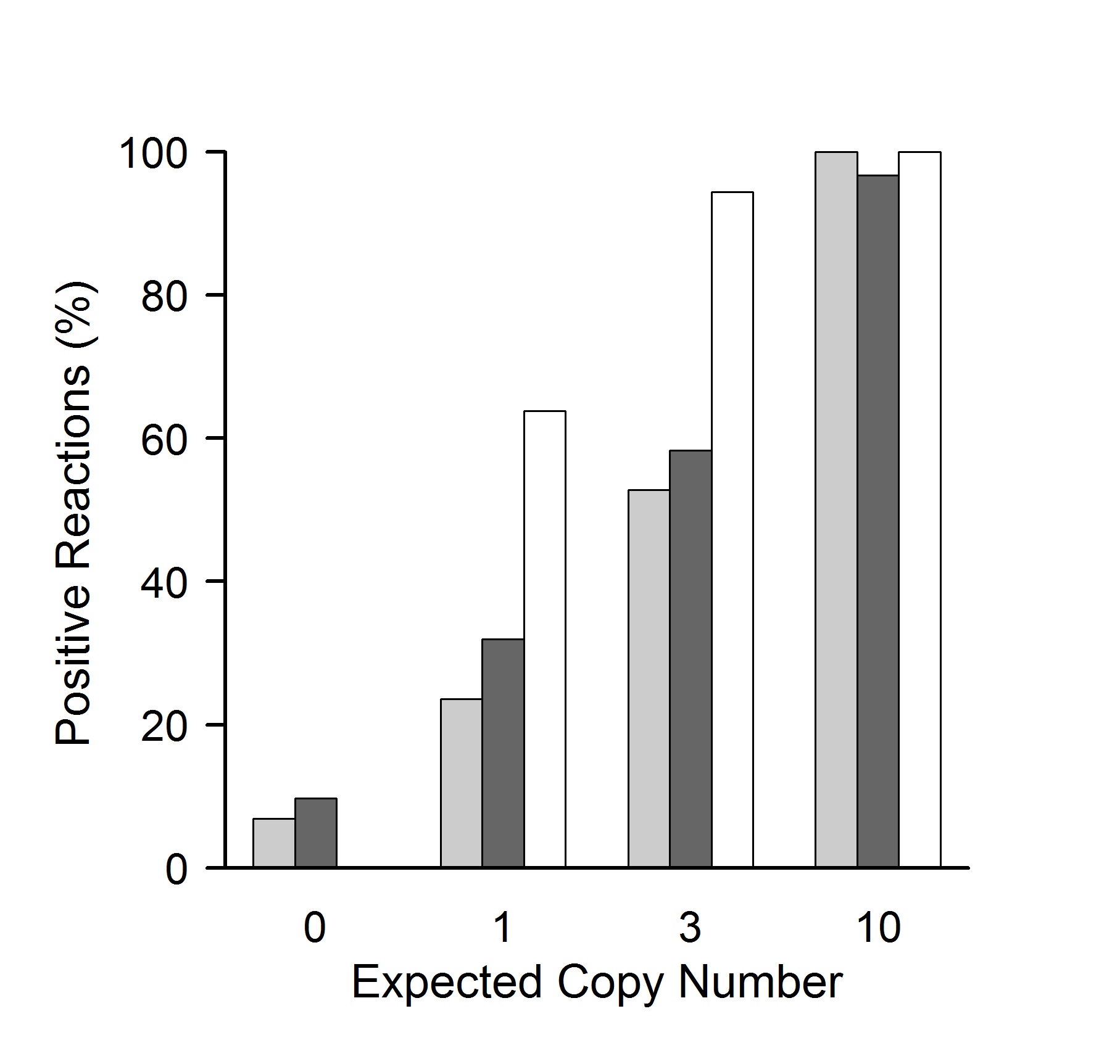

Supplement: S11 Fig — At very low copy numbers, only part of the reaction wells can contain the target gene due to Poisson distribution. Therefore, even under ideal conditions in less than 100% of the reaction wells target DNA can be detected (see also Fig 2). S11 Fig shows the relation between spiked copies (x-axis) and the percentage of positive reactions (y-axis). White bars represent the percentage of reactions that are expected to yield positive signals following ideal Poisson distribution. Light and dark grey columns represent the percentage of reactions that yielded positive signals for PTEN A167T detection in a background of 105 and 2 × 10^5^ wild type copies respectively. Reaction numbers: see S9 Fig for details on qPCR plate layout see Fig 2. While at 10 copies per reaction the number of positive wells nearly represents ideal conditions, at 1 and 3 copies the assay detects less than expected positive samples. Reduction of positive calls below 10 starting copies is common in PCR based methods, even without the demanding conditions of mutation detection [49]. Under the extreme sensitivity and specificity constraints tested, the performance of this assay, i.e. correct calling of on average one single mutation per reaction in 2 × 10^5^ wild type DNAs, is unprecedented in qPCR. The reduction of positive calls at very low copy number is the trade-off for extreme specificity. As shown, it can be compensated by the possibility apply multiple wells per run. The PTEN A167T assay correctly detects and differentiates between 0, 1, 3 and 10 spiked copies both against a background of 105 and 2 × 10^5^ wild type copies in our setting (S9 Fig). (TIF) [file pone.0142273.s011.tif]
